# Ana1/Cep295 regulates centriole elongation via Cep135 and microtubules

**DOI:** 10.1083/jcb.202504094

**Published:** 2026-07-30

**Authors:** Zhenjie Wang, Yuxuan Qian, Xuan Wang, Qianyu Ng, Hanxi Zhang, Yang Wu, Zhen Liu, Zhe Feng

**Affiliations:** 1 State Key Laboratory of Genetics and Development of Complex Phenotypes, School of Life Sciences & Zhongshan Hospital, Fudan University, Shanghai, China; 2 Department of Life Science, The Hong Kong University of Science and Technology, Hong Kong Special Administrative Region, China

## Abstract

Centrioles are essential for centrosome and cilium formation. In fly spermatocytes, they undergo dramatic elongation to form giant centrioles that support sperm development. The conserved centriole protein Ana1/Cep295 is known to promote this elongation, yet the underlying mechanism remains poorly understood. Here, we show that Ana1 regulates centriole length through two distinct mechanisms. It directly interacts with Cep135 to promote centriole elongation and to recruit Cep135 into the proximal centriole-like structure. Ana1 also binds and bundles microtubules *in vitro*, and this activity is required for centriole elongation *in vivo*. Importantly, these two interactions are not mutually exclusive and both contribute to Ana1-induced centriole overelongation. Together, our findings reveal that Ana1 is involved in centriole elongation through interactions with distinct centriolar components during fly spermatogenesis.

## Teaser

Wang et al. uncovered the molecular basis of Ana1-driven centriole elongation in *Drosophila* spermatogenesis. They demonstrated that Ana1/CEP295 promotes longitudinal centriole extension through direct interactions with both Cep135 and microtubules, providing new mechanistic insights into how giant centrioles are assembled during sperm development.

## Introduction

Centrioles are microtubule (MT)-based structures that are essential for the assembly of centrosomes and cilia/flagella (Azimzadeh, 2014). Centrosomes function as the primary MT-organizing centers in most animal cells and play pivotal roles in a variety of cellular processes, including cell division, cell differentiation, and the establishment of cell polarity (Bettencourt-Dias et al., 2011; Conduit et al., 2015; Nigg and Raff, 2009). In quiescent cells that enter the G_0_ phase, centrioles dock at the plasma membrane where they become basal bodies to initiate the formation of cilia and flagella (Dutcher, 2003). Defects in centriole assembly result in the malformation and dysfunction of these critical organelles, and have been linked to a diverse set of human pathologies. Therefore, elucidating mechanisms that govern centriole biogenesis and maintenance is crucial for understanding their function and the pathological consequences of their dysfunction.

Centriole assembly begins with the recruitment of the protein kinase ZYG1 (in worms) or Plk4 (in mammals and flies) to the outer wall of the mother centriole (Bettencourt-Dias et al., 2005; Habedanck et al., 2005; O’Connell et al., 2001; Sonnen et al., 2012), which triggers the hierarchical assembly of core cartwheel components including SAS5/STIL/Ana2 (worms, mammals, and flies, respectively) and Sas6 (Arquint et al., 2015; Kratz et al., 2015; Lettman et al., 2013; Moyer et al., 2015; Ohta et al., 2014). The resulting ninefold symmetric cartwheel structure then provides a scaffold for centriole formation (Banterle et al., 2021; Kitagawa et al., 2011; Laporte et al., 2024; van Breugel et al., 2011). Subsequent recruitment of Sas4/CPAP promotes the polymerization of centriolar MTs, which surround and stabilize the inner cartwheel structure (Cottee et al., 2013; Hatzopoulos et al., 2013; Hsu et al., 2008; Tang et al., 2009; Tang et al., 2011).

Following their assembly, centrioles recruit pericentriolar material (PCM) and mature into centrosomes. Recent studies have demonstrated that the recruitment of Cep295 (human)/Ana1 (*Drosophila*) is essential for centriole-to-centrosome conversion (Fu et al., 2016; Izquierdo et al., 2014; Tsuchiya et al., 2016). In human cell lines, depletion of Cep295 impairs the recruitment of PCM and leads to centriole destabilization (Izquierdo et al., 2014). In *Drosophila*, *ana1* mutant flies are severely uncoordinated due to the lack of functional cilia and exhibit a significantly reduced number of centrosomes in third-instar larval brains (Blachon et al., 2009; Saurya et al., 2016). In cultured fly cell lines, Ana1 is recruited to centrioles during late anaphase and is required for the recruitment of Asl (Fu et al., 2016), which in turn promotes PCM recruitment and the subsequent maturation of centrioles into centrosomes. Asl also licenses the daughter centriole for duplication in the next cell cycle through the recruitment of the Plk4 kinase (Novak et al., 2014; Novak et al., 2016). In addition, Ana1 contributes to centrosome maintenance in oocytes, a function that depends on Polo kinase but is independent of the PCM (Pimenta-Marques et al., 2024).

Apart from its role in centriole-to-centrosome conversion, Ana1 has also been implicated in centriole elongation, particularly in the male germline where centrioles undergo dramatic growth during spermatogenesis (Alvarez-Rodrigo et al., 2021; Saurya et al., 2016). In fly spermatocytes, the centriole length is directly correlated with the expression level of Ana1: Ana1 overexpression induces the overelongation of centrioles, whereas reduced Ana1 dosage leads to the formation of shorter centrioles. However, the molecular mechanism underlying this function of Ana1 remains poorly understood. Here, we identified distinct regions within Ana1 that mediate interactions with Cep135 and MTs, and demonstrated that both activities are required for centriole length control. We further showed that the Ana1-Cep135 interaction is dispensable for the recruitment of Ana1 to centrioles, but is specifically required for loading Cep135 into the proximal centriole-like (PCL) structure during later stages of spermatogenesis, suggesting a context-dependent Ana1-Cep135 recruitment hierarchy.

## Results

### Ana1 is essential for centriole assembly and promotes centriole overelongation in fly spermatocytes

**Figure 1.**
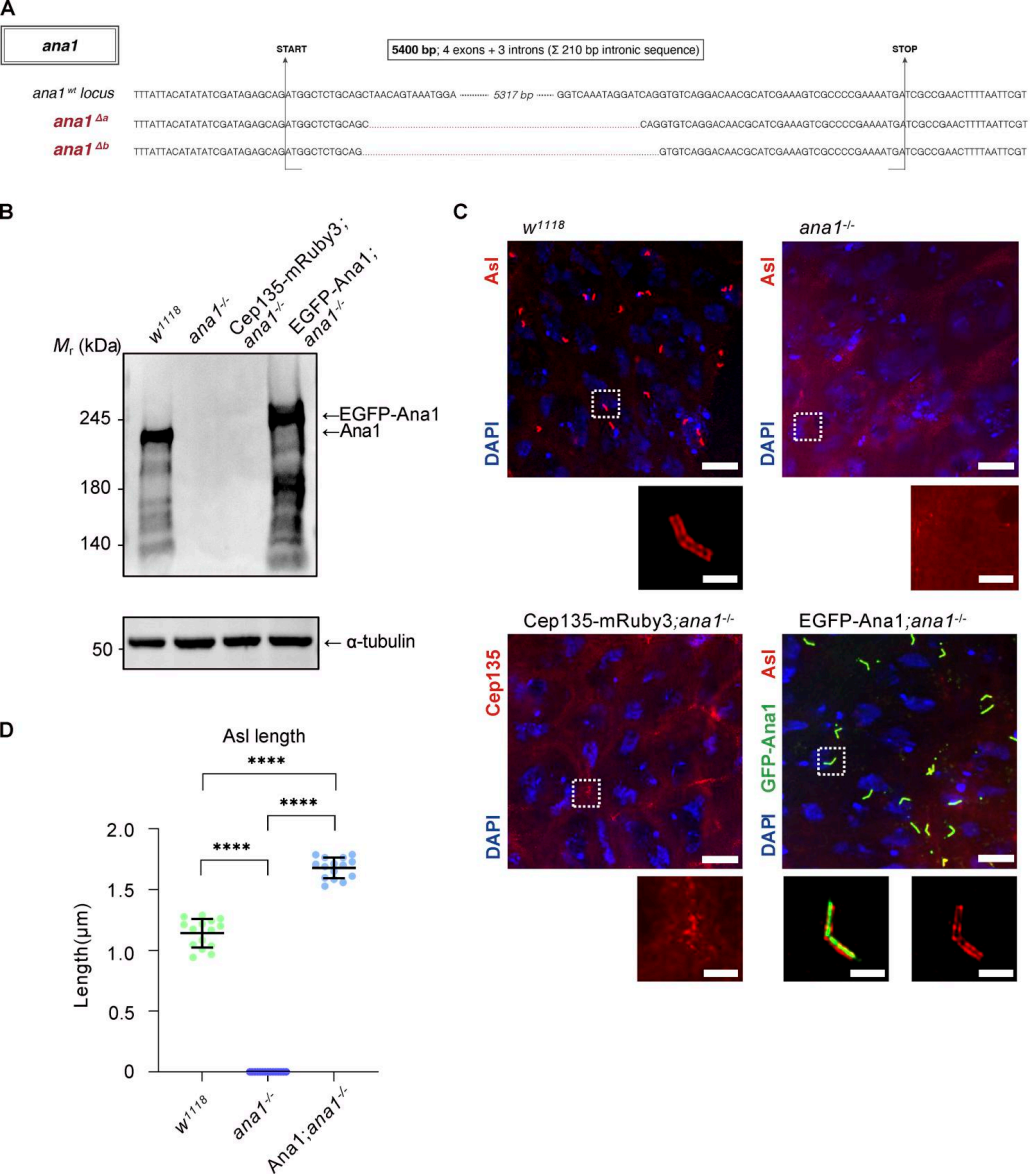
**Ana1 is essential for centriole duplication and promotes centriole overelongation in fly spermatocytes. (A)** Schematic representation of the two independent *ana1* null alleles, denoted as *ana1*^*Δa*^ and *ana1*^*Δb*^, created by CRISPR/Cas9 mutagenesis. Both mutants had deletion of the entire *ana1* coding region. These alleles were then crossed to generate the *ana1* null mutant background (referred to as *ana1*^*−/−*^) used throughout this study. **(B)** Western blot of extracts from 50 to 60 testes of indicated genotypes. Detection of Ana1 was performed using specific antibodies, with α-tubulin as a loading control for protein levels in lysates. **(C)** Centrioles in primary spermatocytes stained to reveal EGFP-Ana1 (green), Asl (red), Cep135-mRuby3 (red), and DNA (blue). Scale bars, 10 µm (overview image) or 2 µm (insets). **(D)** Graphs show the mean ± SD quantification for centriole lengths in mature primary spermatocytes, using Asl as a centriole marker for each genotype. Each point on the graph represents the average length measured from a 16-cell cyst with 10–15 centrioles scored; five independent testes were measured for each genotype with 2–3 cell cysts scored in each testis. ****P < 0.0001. Source data are available for this figure: SourceData F1.

Previous studies of Ana1’s function in centriole and centrosome assembly have relied largely on the *ana1*^*mecB*^ mutant. However, recent work has suggested that the truncated Ana1 protein encoded by this mutant allele may confound phenotypic interpretation through intragenic complementation (Nagy et al., 2025). To reexamine the functional role of Ana1 in fly spermatocytes, we therefore analyzed a CRISPR/Cas9-derived *ana1* null mutant that lacks the entire coding region, including all four exons and three introns (Fig. 1, A and B; see *Materials and methods* for details). Unless otherwise stated, all experiments in this study were performed using this null mutant.

**Figure S1.**
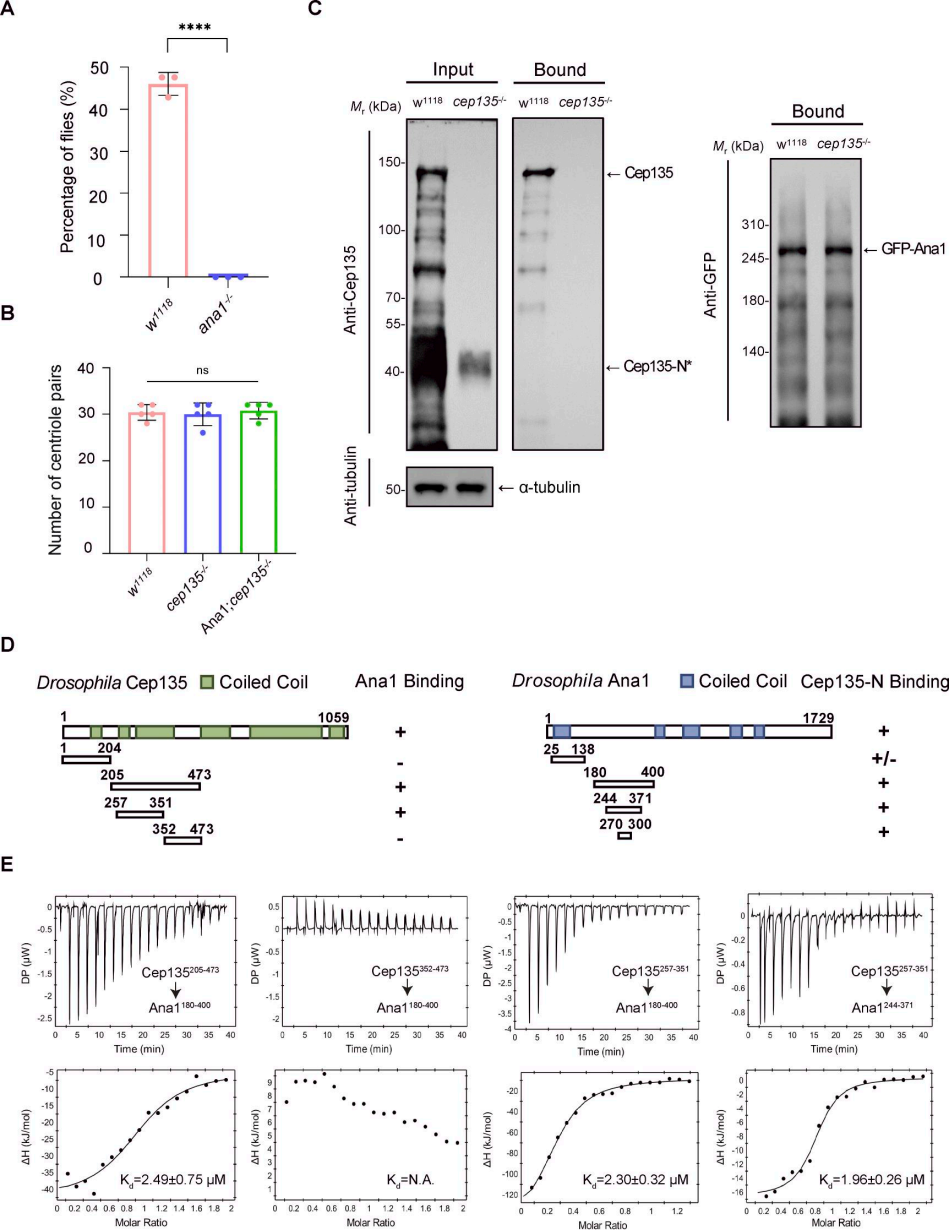
**Mapping of the interaction region between Cep135 and Ana1. (A)** Climbing ability was assessed by measuring the percentage of flies per genotype that climbed past the 5-cm mark within 2 s. Each data point represents the percentage from 20 flies, with three independent biological replicates using distinct cohorts of flies per genotype (total of 60 flies per genotype). Error bars indicate the mean ± SD. ****P < 0.0001 (unpaired two-tailed Student’s *t* test). **(B)** Centriole pairs, marked by Asl localization, were quantified in 15–20 cells per testis from the indicated genotypes. Each data point represents the number of centriole pairs per testis, derived from five independent testes per genotype. Error bars indicate the mean ± SD. ns, not significant (unpaired two-tailed Student’s *t* test). **(C)** Pull-down of EGFP-Ana1 from S2 cells incubated with testis lysates (150 testes/genotype) from *w*^1118^ or *cep135*^c04199^ mutants. Blots were probed with anti-Cep135 (aa1–225). α-Tubulin is a loading control. The residual Cep135 in mutants (Cep135-N*) fails to bind Ana1. **(D and E)** ITC-based experiments to screen for interactions between Cep135 and Ana1 fragments, and a schematic representation showing various Ana1 and Cep135 constructs analyzed for binding in this study. Source data are available for this figure: SourceData FS1.

*ana1* mutant flies were severely uncoordinated, likely due to defective centriole duplication and the consequent failure to form functional cilia in mechanosensory neurons (Fig. S1 A) (Blachon et al., 2009). In *ana1*^*−/−*^ spermatocytes, Asl localization was completely abolished, consistent with its known dependency on Ana1 for centriolar recruitment (Fig. 1 C). Although Cep135-mRuby3–positive foci were occasionally observed, no morphologically recognizable centrioles could be detected (Fig. 1 C). These defects were fully rescued by the expression of EGFP-Ana1 under the control of a ubiquitin promoter (Fig. 1, B and C). Moreover, as previously reported, the overexpression of EGFP-Ana1 induced centriole overelongation in spermatocytes (Fig. 1, C and D). Together, our data confirmed that Ana1 is essential for centriole assembly *in vivo* and also established a robust genetic system to dissect mechanisms by which Ana1 promotes centriole elongation in fly spermatocytes.

### Cep135 is required for Ana1-induced centriole overelongation

**Figure 2.**
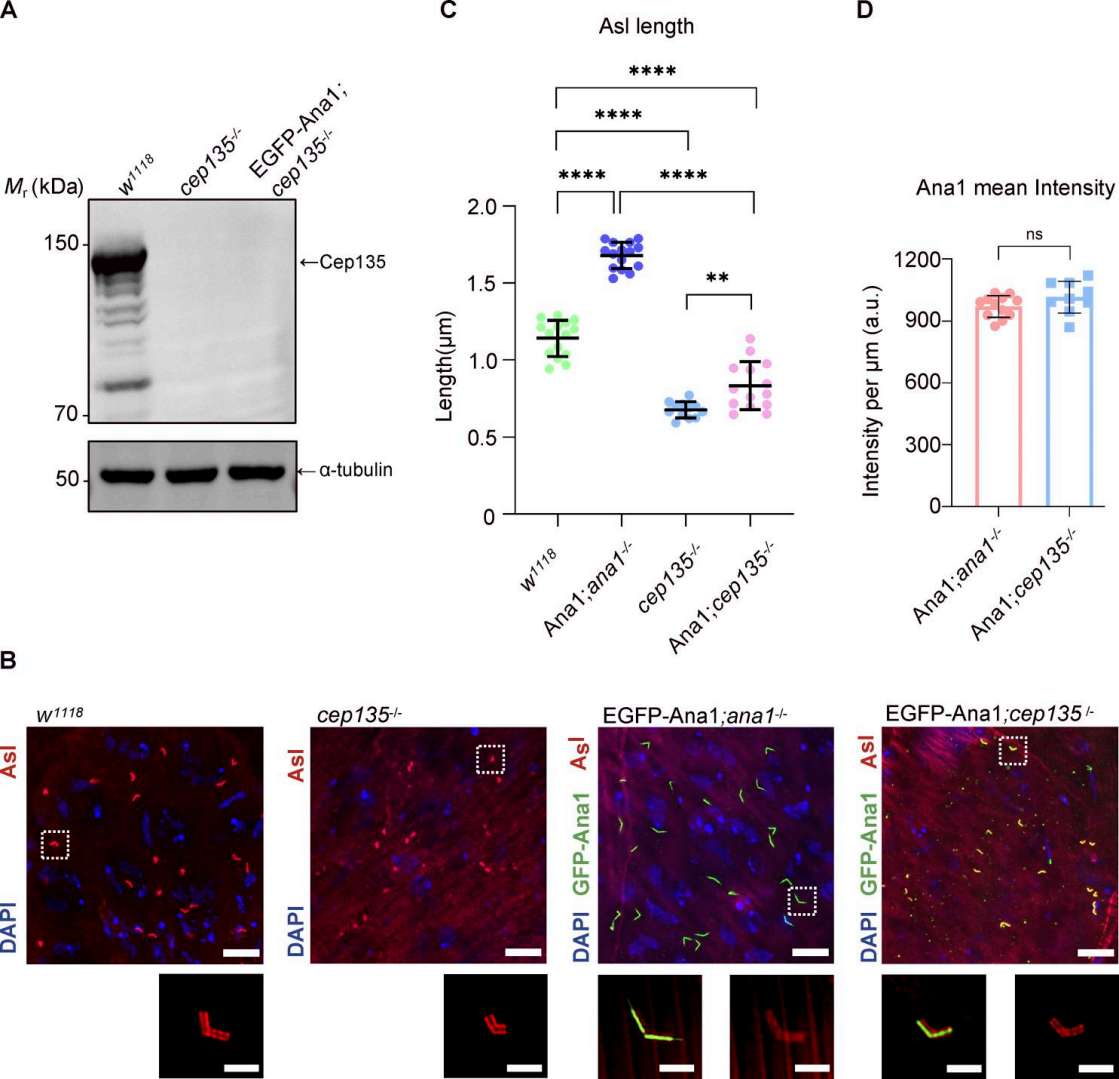
**Cep135 is required for Ana1-induced centriole overelongation. (A)** Western blot of extracts from 50 to 60 testes of indicated genotypes. Detection of Cep135 was performed using specific antibodies, with α-tubulin as a loading control for protein levels in lysates. **(B)** Centrioles in primary spermatocytes stained to reveal EGFP-Ana1 (green), Asl (red), and DNA (blue). Scale bars, 10 µm (overview image) or 2 µm (insets). **(C)** Graphs show the mean ± SD quantification for centriole lengths in mature primary spermatocytes, using Asl as a centriole marker for each genotype. Each point on the graph represents the average length measured from a 16-cell cyst with 10–15 centrioles scored; five independent testes were measured for each genotype with 2–3 cell cysts scored in each testis. ****P < 0.0001, **P < 0.01. **(D)** Graphs show the mean ± SD quantification of Ana1 mean fluorescence intensity (per unit length of centriole) for each genotype. Each point on the graph represents the average intensity measured from a 16-cell cyst with 10–15 centrioles scored; five independent testes were measured for each genotype with 2–3 cell cysts scored in each testis. ns, not significant using a two-tailed unpaired *t* test. Source data are available for this figure: SourceData F2.

Previous studies have shown that loss of Cep135 leads to shorter centrioles and the absence of the central pair of MTs in flagellar axonemes (Blachon et al., 2009; Carvalho-Santos et al., 2012; Mottier-Pavie and Megraw, 2009; Roque et al., 2012). To determine whether Cep135 is required for Ana1-dependent centriole overelongation, we expressed EGFP-Ana1 in *cep135*^*c04199*^ mutant flies. Depletion of full-length Cep135, confirmed by western blotting (Fig. 2 A), strongly suppressed Ana1-induced centriole overelongation in primary spermatocytes, although these centrioles were marginally longer than those in *cep135*^*c04199*^ flies without Ana1 overexpression (Fig. 2, B and C). Consistent with previous reports (Blachon et al., 2009; Roque et al., 2012), centriole duplication appeared largely unaffected (Fig. S1 B). Notably, EGFP-Ana1 still localized robustly to centrioles in the absence of Cep135 (Fig. 2 B), and the amount of Ana1 recruited per unit length of centrioles was unchanged (Fig. 2 D). Since the *cep135*^*c04199*^ mutant is predicted to retain an N-terminal fragment (Fu et al., 2016; Mottier-Pavie and Megraw, 2009), we considered whether this residual protein might facilitate Ana1 recruitment. Pull-down experiments demonstrated that this fragment does not interact with Ana1 (Fig. S1 C). Taken together, these results indicate that Cep135 is required for Ana1-induced centriole overelongation but is dispensable for the initial recruitment of Ana1 to centrioles in fly spermatocytes.

### Mapping of the Ana1-Cep135 interaction interface

**Figure 3.**
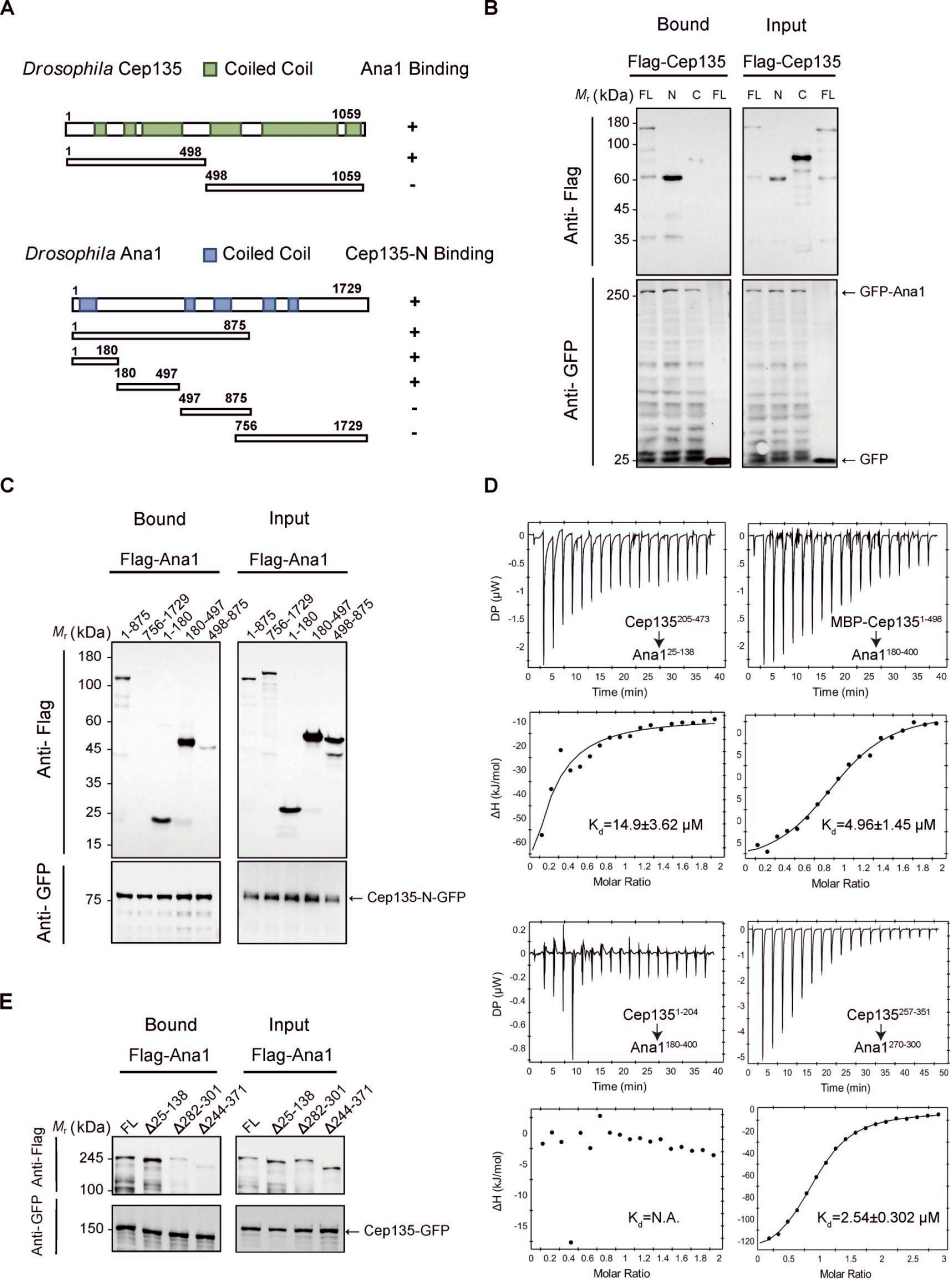
**Ana1 binds to Cep135 via two distinct interfaces. (A)** Schematic representation of the various Ana1 or Cep135 deletion constructs analyzed in this study. The N-terminal half of Cep135 (aa1–498, referred to as Cep135-N) binds to Ana1, whereas the C-terminal half (aa498–1059, referred to as Cep135-C) does not, while for Ana1, the N-terminal fragments aa1–180 and aa180–497 both bind to Cep135-N, but the C-terminal half does not. **(B)** S2 cells were transiently cotransfected with GFP-tagged Ana1 and Flag-tagged Cep135 fragments. Extracts were then subjected to pull-down experiments using GFP beads, and bound fractions were analyzed by western blotting. The experiment was repeated three times with similar results. **(C)** S2 cells were transiently cotransfected with GFP-tagged Cep135-N and Flag-tagged Ana1 fragments. Extracts were then subjected to pull-down experiments using GFP beads, and bound fractions were analyzed by western blotting. The experiment was repeated three times with similar results. **(D)** ITC-based experiments showing direct interaction between Cep135 and Ana1. 400 μM Cep135 fragments in the syringe were titrated into 40 μM Ana1 fragments in the cell. The experiment was repeated three times, and the average K_d_ value is displayed. **(E)** S2 cells were transiently cotransfected with GFP-tagged Cep135 and Flag-tagged Ana1, which carries deletion of the two identified binding regions. Extracts were then subjected to pull-down assays using GFP beads and were analyzed by western blotting. The experiment was repeated three times with similar results. Source data are available for this figure: SourceData F3.

To determine whether Cep135 contributes to Ana1-induced centriole overelongation through a direct interaction, we set out to define the binding interface between the two proteins. Although Ana1 has previously been shown to interact with Cep135 in cultured D.Mel-2 cells (Fu et al., 2016), the underlying interface has remained poorly characterized. We therefore performed GFP pull-down experiments using a series of Ana1 and Cep135 fragments (Fig. 3 A). Consistent with previous findings, the full-length Ana1 protein showed robust binding to the N-terminal region of Cep135 (Fig. 3 B). Further mapping experiments revealed that Cep135 binds two distinct regions within Ana1, aa1–180 and 180–497 (Fig. 3 C).

We next measured the binding affinity between Cep135 and Ana1 by isothermal titration calorimetry (ITC)–based experiments using recombinantly purified MBP-tagged Cep135^1–498^ and the two fragments of Ana1 (aa25–138 and aa180–400) (Fig. 3 D). Cep135^1–498^ formed a 1:1 complex with Ana1^180–400^, with a dissociation constant (K_d_) of ∼4.96 μm, whereas its interaction with Ana1^25–138^ was weaker (K_d_ of ∼14.9 μm). Additional ITC-based measurements using shorter fragments of both proteins further identified Cep135^257–351^ and Ana1^270–300^ as the minimal binding regions, with a K_d_ of ∼2.54 μm (Fig. 3 D; and Fig. S1, D and E).

To evaluate the contribution of these two Ana1 regions to Cep135 binding, we made deletion constructs targeting each mapped interaction site and performed GFP pull-down assays. Deletion of the major Cep135-binding region (aa282–301/aa244–371) nearly abolished the interaction, whereas deletion of the weaker N-terminal region (aa25–138) had no detectable effect (Fig. 3 E). It should be noted that this weaker N-terminal region overlaps with the highly conserved CR1 domain (aa19–134), which is found in all CEP295/Ana1 homologs (Tsuchiya et al., 2016). Thus, although dispensable for Cep135 binding, its strong evolutionary conservation suggests that it may have other functional roles.

**Figure 4.**
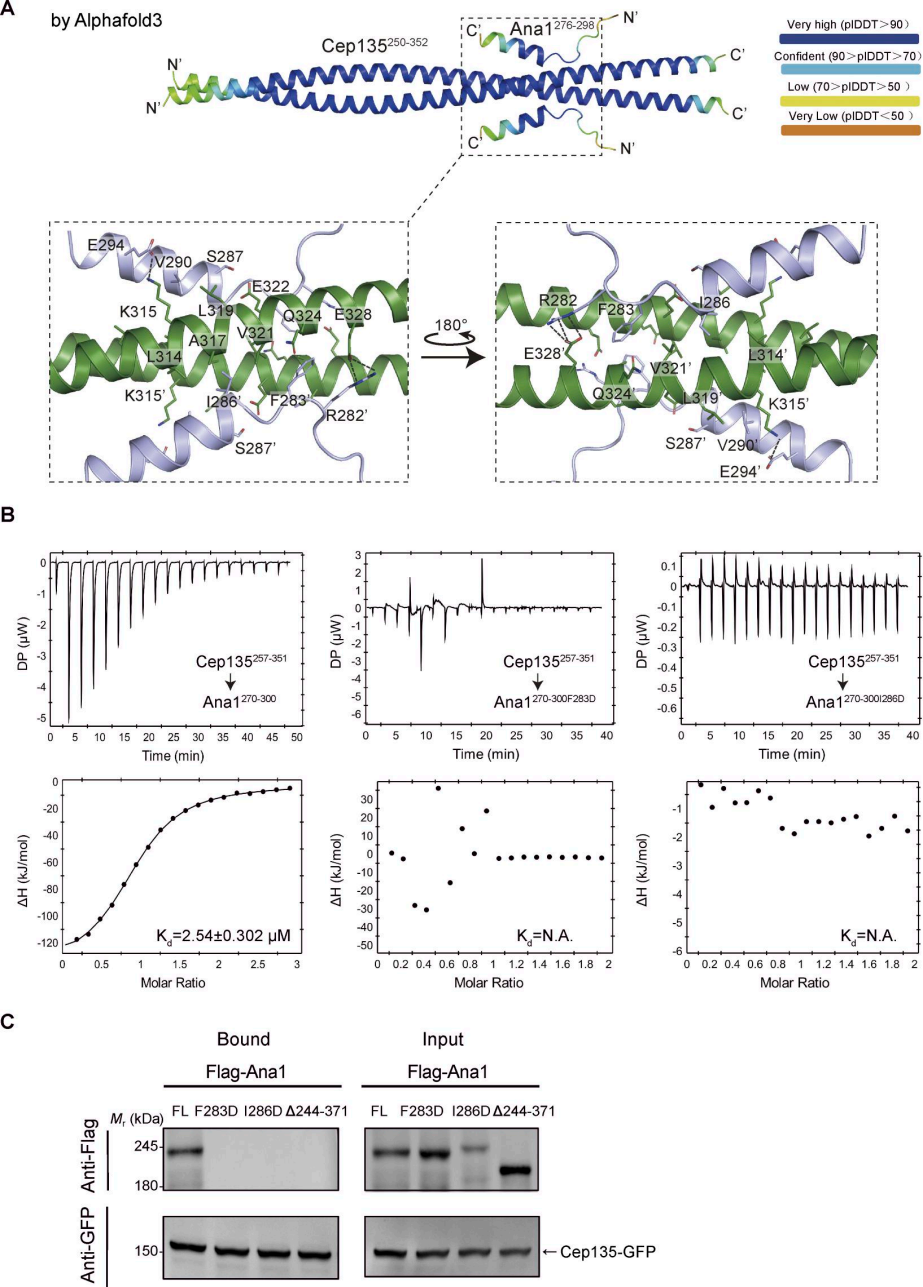
**Identification of key residues within Ana1 that bind Cep135. (A)** Cartoon representation for the structural model of the Cep135^250–352^-Ana1^276–298^ complex predicted by AF3-based analysis. Underneath are the zoomed-in views showing residues at the protein-binding interface. Dotted lines indicate potential electrostatic interactions formed between conserved residues within Ana1 and Cep135. **(B)** ITC experiments showing substitution of the conserved, core hydrophobic residues at the binding interface with negatively charged Asp residues completely abolished the Cep135-Ana1 interaction. The experiment was repeated three times, and the average K_d_ value is displayed. **(C)** S2 cells were transiently cotransfected with Flag-tagged Ana1 mutants and GFP-tagged Cep135. Extracts were then subjected to pull-down assays using GFP beads and were analyzed by western blotting. The experiment was repeated three times with similar results. Source data are available for this figure: SourceData F4.

**Figure S2.**
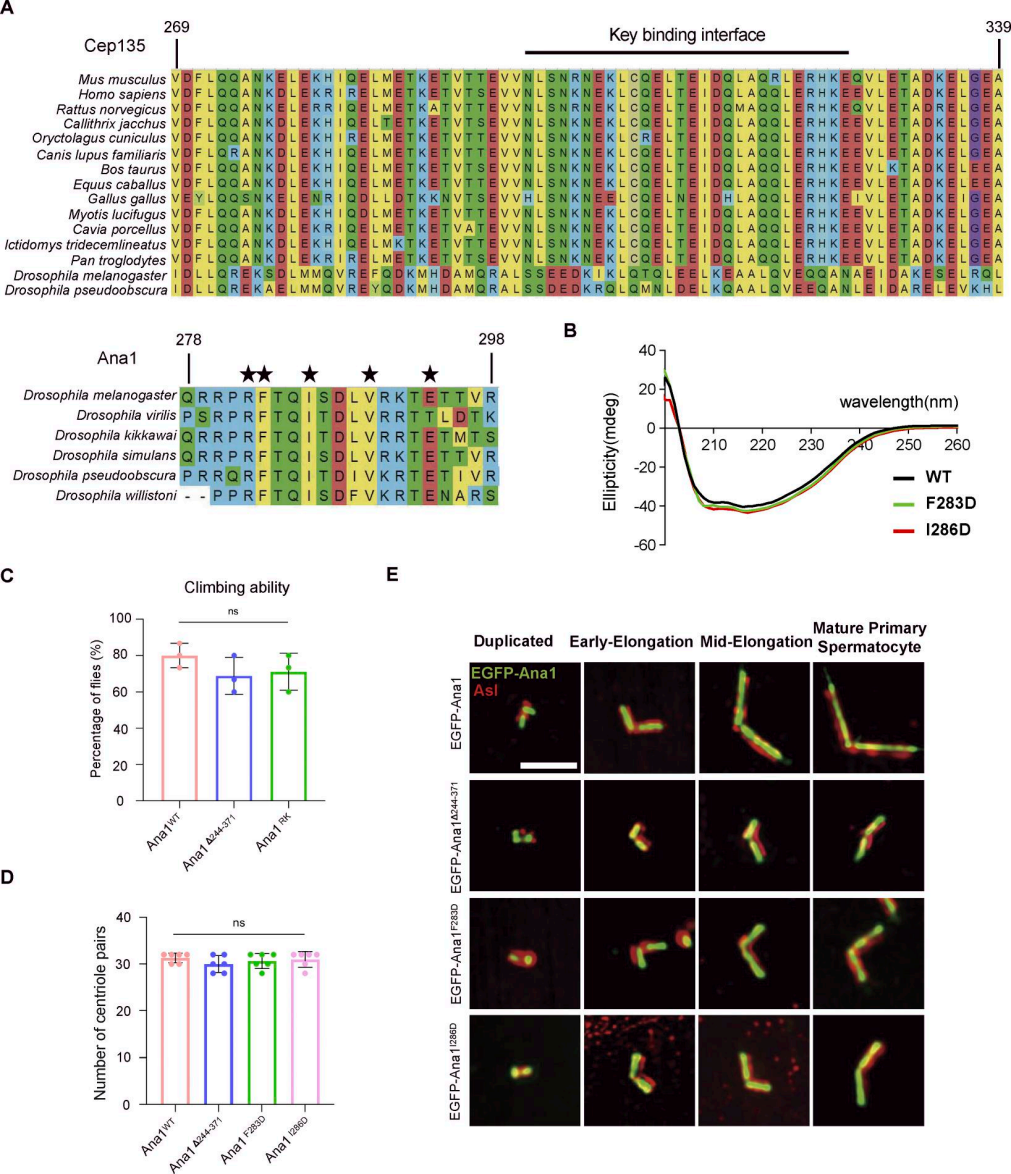
**Cep135 binding–deficient Ana1 mutants fail to induce centriole overelongation. (A)** MSA showing the conservation of residues involved in the Cep135-Ana1 interaction. Note that sequences of Ana1/Cep295 homologs are less well conserved through evolution, and thus, an MSA was performed using *Drosophila* sequences. **(B)** CD analysis showing that WT Ana1 and Ana1 mutant proteins are all largely helical in nature. **(C)** Climbing ability was assessed by measuring the percentage of flies per genotype that climbed past the 5-cm mark within 2 s. Each data point represents the percentage from 15 flies, with three independent biological replicates using distinct cohorts of flies per genotype (total of 45 flies per genotype). Error bars indicate the mean ± SD. ns, not significant (unpaired two-tailed Student’s *t* test). **(D)** Centriole pairs, marked by Asl localization, were quantified in 15–20 cells per testis from the indicated genotypes. Each data point represents the number of centriole pairs per testis, derived from five independent testes per genotype. Error bars indicate the mean ± SD. ns, not significant (unpaired two-tailed Student’s *t* test). **(E)** Representative images showing centriole elongation in primary spermatocytes at various stages from different genotypes, stained for EGFP-Ana1 (green) and Asl (red). Scale bar, 2 µm. WT, wild type; CD, circular dichroism; MSA, multiple sequence alignment.

**Figure S3.**
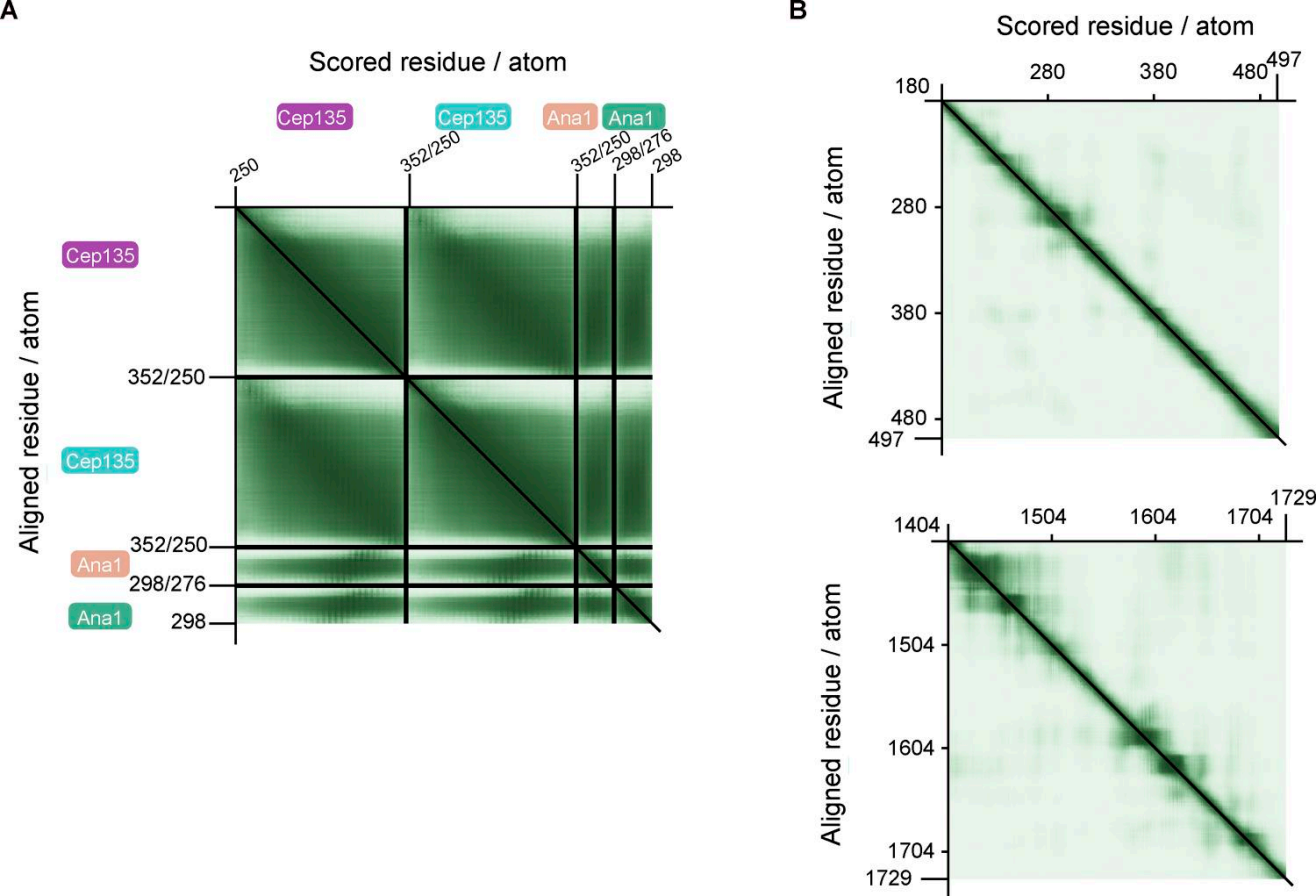
**PAE plots for AF3-predicted structures. (A and B)** PAE plots for the AF3-predicted structure of the Ana1-Cep135 complex (A) and the Ana1 apo (B). PAE is a confidence measure for the relative position of any two residues within the predicted structure. Darker green colors indicate higher confidence levels. PAE, Predicted Aligned Error.

Having defined the major Cep135-binding region in Ana1, we next sought to gain structural insights into this interaction. We used AlphaFold3 (AF3)-based prediction (Abramson et al., 2024) to model the Cep135^250–352^-Ana1^276–298^ complex (Fig. 4 A). The five top-ranked solutions predicted highly similar models of a heterotetrameric complex, which is composed of an Ana1 dimer and a Cep135 dimer arranged in an antiparallel orientation (pTM = 0.66, ipTM = 0.65; Fig. S3 A). Evolutionarily conserved residues were enriched at the packing interface between the two proteins (Fig. 4 A and Fig. S2 A). In particular, the invariant residues Phe283, Ile286, and Val290 of Ana1 were buried within the hydrophobic core of the coiled-coil tetramer, while the invariant Arg282 and the highly conserved Glu294 residues were positioned to form electrostatic interactions with the adjacent Cep135 dimer.

To test the potential relevance of these predicted contacts, we substituted the two core hydrophobic residues in Ana1 with charged aa (F283D and I286D). Neither substitution appeared to drastically perturb the folding of Ana1 *in vitro* (Fig. S2 B), but both completely abolished Cep135 binding, as demonstrated by ITC-based experiments and GFP pull-down assays (Fig. 4, B and C). Together, these data strongly support the predicted contact interface between Ana1 and Cep135.

### The Ana1-Cep135 interaction promotes centriole elongation

**Figure 5.**
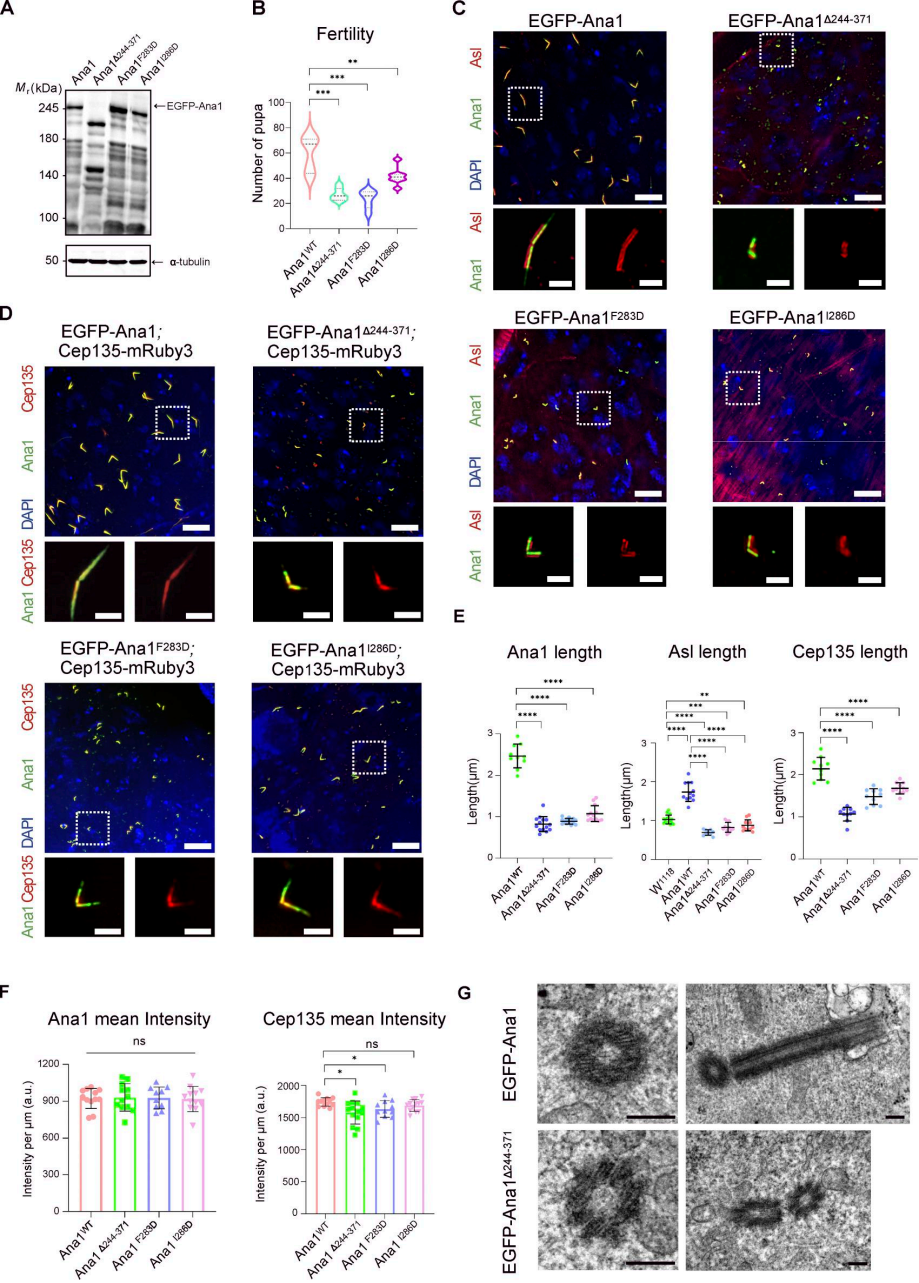
**Ana1-Cep135 interaction is essential for centriole length control in primary spermatocytes. (A)** Western blot of extracts from 50 to 60 testes of indicated genotypes. Immunoblot analysis of Ana1 protein variants with an anti-Ana1 primary antibody. α-Tubulin is shown as a loading control. **(B)** Graphs showing the male fertility of different genotypes, as measured by the number of progeny pupae produced from mating with WT (*w*^*1118*^) virgin females. **P < 0.01, ***P < 0.001. **(C and D)** Centrioles in primary spermatocytes stained to reveal EGFP-Ana1 (green), Asl (red), Cep135-mRuby3 (red), and DNA (blue). Scale bar, 10 µm (overview image) or 2 µm (insets). **(E)** Graphs showing the mean ± SD quantification for centriole lengths in mature primary spermatocytes, using Ana1, Asl, or Cep135 as a centriole marker for each genotype. Each point on the graph represents the average length measured from a 16-cell cyst with 10–15 centrioles scored; five independent testes were measured for each genotype with 2–3 cell cysts scored in each testis. **P < 0.01, ***P < 0.001, ****P < 0.0001. **(F)** Graphs showing the mean ± SD quantification of Ana1 or Cep135 mean fluorescence intensity (per unit length of centriole) for each genotype. Each point on the graph represents the average mean intensity measured from a 16-cell cyst with 10–15 centrioles scored; five independent testes were measured for each genotype with 2–3 cell cysts scored in each testis. *P < 0.05. ns, not significant. **(G)** TEM of EGFP-Ana1 (top panels) and EGFP-Ana1^Δ244–371^ (bottom panels) in cross-sections (left) and longitudinal sections (right) of centrioles. Cross-sectional views reveal that the ninefold symmetric centriole architecture is preserved in both WT and mutant flies analyzed. Longitudinal sections show that full-length EGFP-Ana1 labels normal-length centrioles (top right), whereas deletion of the residues 244–371 results in shortened centrioles (bottom right). For each genotype, centrioles were examined from at least three independent testes, with a total of 8–12 centrioles analyzed. Scale bar, 200 nm. WT, wild type. Source data are available for this figure: SourceData F5.

In order to investigate the functional importance of the Ana1-Cep135 interaction *in vivo*, we generated transgenic flies expressing either WT EGFP-Ana1 or Cep135 binding–deficient Ana1 mutants under the control of a ubiquitin promoter. These included a deletion mutant lacking the major Cep135-binding region (Ana1^Δ244–371^) and two single-point mutants, Ana1^F283D^ and Ana1^I286D^, both of which abolished Cep135 binding *in vitro*. Western blotting confirmed that WT and mutant Ana1 proteins were expressed at similar levels (Fig. 5 A). Both EGFP-Ana1 and its binding-deficient mutants strongly rescued the severe uncoordination defect of *ana1* mutant flies (Fig. S2 C), and all rescued flies were fertile. However, male flies that expressed the Cep135 binding–deficient Ana1 mutants produced significantly fewer pupae (Fig. 5 B). These results suggest that the Ana1-Cep135 interaction is dispensable for neurosensory cilium function but critical for germline function, particularly during spermatogenesis.

We next analyzed the behavior of WT and mutant EGFP-Ana1 proteins and their effects on centriole assembly in primary spermatocytes. All EGFP-Ana1 variants localized to centrioles marked by Asl, and centriole duplication appeared unaffected (Fig. 5 C and Fig. S2 D). The Cep135-mRuby3 signal was also detected along the entire centriole length in all genotypes (Fig. 5 D). Nevertheless, mutant centrioles were significantly shorter than WT controls and, notably, even shorter than those assembled from endogenous Ana1 (Fig. 5 E and Fig. S2 E). Thus, the Ana1-Cep135 interaction is required not only for Ana1-induced centriole overelongation but also for normal centriole length control. Quantification of Ana1 and Cep135 levels at centrioles revealed that Ana1 fluorescence intensity per unit length of centrioles was unchanged in all mutants, while Cep135 intensity was slightly reduced (Fig. 5 F). Transmission electron microscopy (TEM) analysis on preparations of WT and mutant testes further confirmed that despite their reduced lengths, no obvious structural defects were detected in the mutant centrioles analyzed (Fig. 5 G). Together, these findings demonstrate that the Ana1-Cep135 interaction is crucial for centriole overelongation and normal centriole length control, but is dispensable for the initial recruitment of Ana1 to centrioles.

### The Ana1-Cep135 interaction promotes Cep135 incorporation into the PCL structure

**Figure 6.**
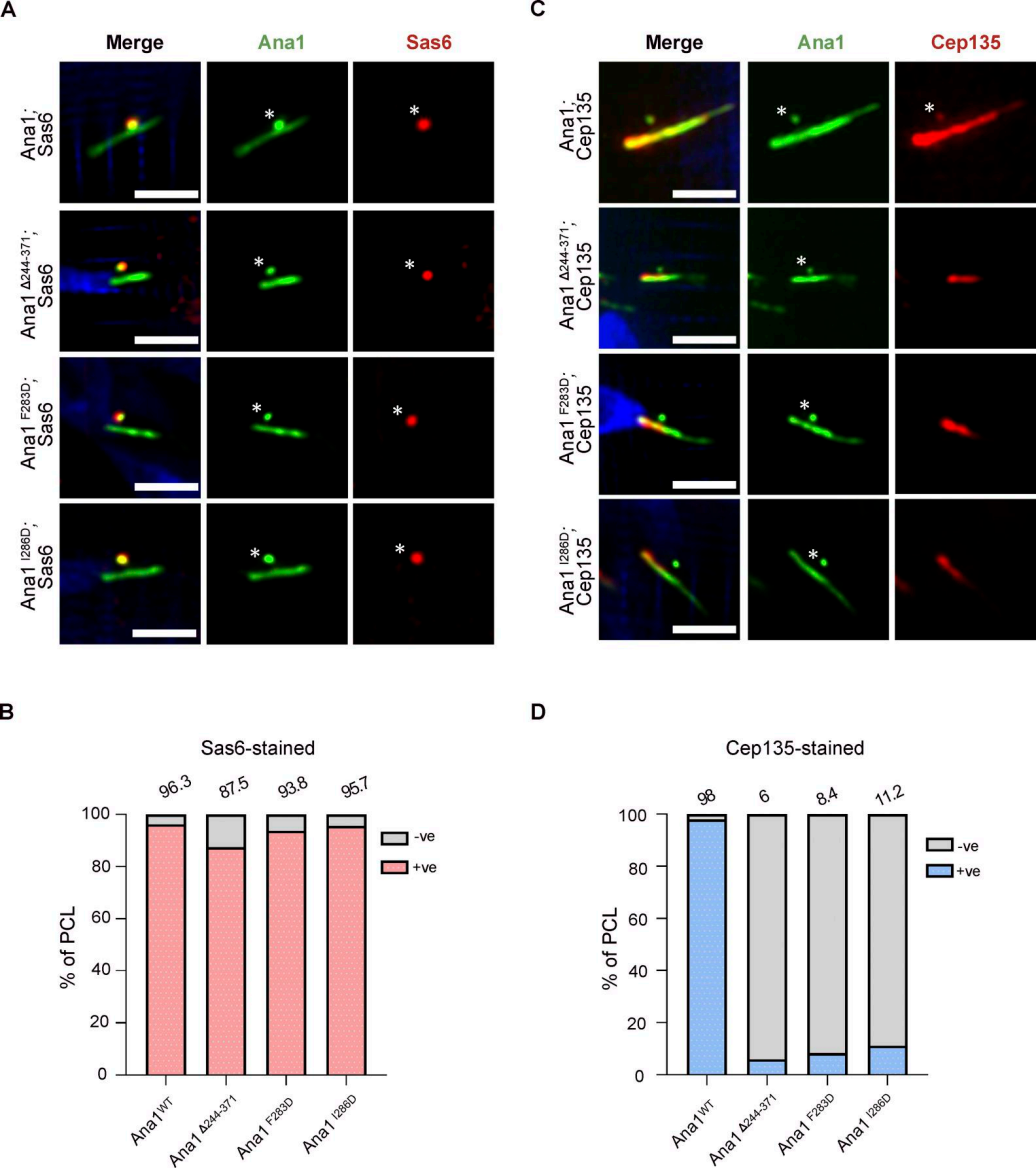
**Ana1-Cep135 interaction is required for the recruitment of Cep135 to the PCL structure. (A)** Representative images of spermatids at the leaf-shaped nucleus stage in indicated genotypes. Colocalization of EGFP-Ana1 (green) with RFP-Sas6 (red) at the PCL structure. Scale bar, 2 µm. **(B)** Quantification of Sas6 localization within the Ana1-positive PCL structure. The percentage of the Sas6-positively stained PCL structure is indicated above each column. Data are from *N* = 80 per genotype collected from five independent testes (*n* = 10–15 PCL structures per testis). **(C)** Representative images of spermatids at the leaf-shaped nucleus stage in indicated genotypes. Colocalization of EGFP-Ana1 (green) with Cep135-mRuby3 (red) at the PCL structure. Scale bar, 2 µm. **(D)** Quantification of Cep135 localization within the Ana1-positive PCL structure. The percentage of Cep135-positively stained PCL structure is indicated above each column. Data are from *N* = 80 per genotype collected from five independent testes (*n* = 10–15 PCL structures per testis).

Having established the role of the Ana1-Cep135 interaction in centriole elongation, we then asked whether this interaction also functions at a later stage of spermiogenesis. In late spermatids, centriole duplication factors are recruited proximal to the base of the centriole/basal body to form the PCL structure. The PCL structure contains core centriole proteins, including Plk4, Ana2, Sas6, Ana1, and Cep135, and serves as a template for daughter centriole formation in the early embryo (Blachon et al., 2009; Buglak et al., 2024). In both WT and mutant spermatids, PCL structures appeared synchronously within the spermatid cyst at the leaf nuclear stage, as marked by an EGFP-Ana1–positive bulge adjacent to one end of the centriole. RFP-Sas6 was robustly recruited to the PCL structure in all genotypes (Fig. 6, A and B). In contrast, Cep135-mRuby3 incorporation into the PCL structure was nearly abolished in spermatids that expressed binding-deficient mutants (Fig. 6, C and D). Thus, the Ana1-Cep135 interaction also contributes to the efficient recruitment of Cep135 into the PCL structure during spermatid maturation.

### Ana1 binds and bundles MTs via its N- and C-terminal regions

Although the Ana1-Cep135 interaction is crucial for centriole overelongation, our earlier finding that Ana1 overexpression induced residual centriole elongation in *cep135*^*−/−*^ spermatocytes (Fig. 2, B and C) raised the possibility that Ana1 may also act through additional mechanisms. Previously published expansion microscopy data have shown that Ana1 localizes to the centriolar wall (Tian et al., 2021), and we therefore speculated that Ana1 may directly associate with MTs.

**Figure 7.**
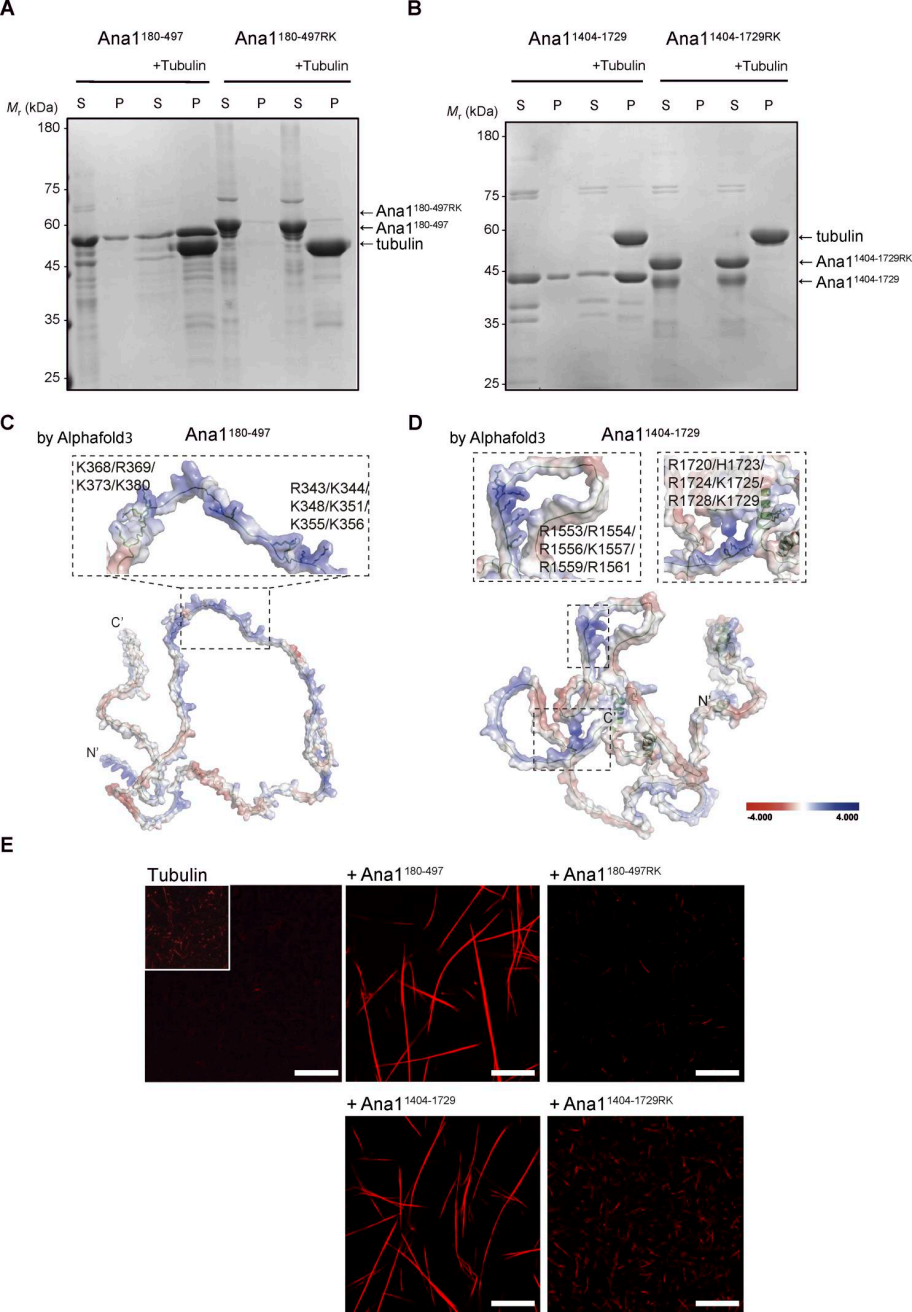
**Ana1 binds to and bundles MTs *in vitro*. (A and B)** Coomassie blue–stained SDS-PAGE gel of a MT cosedimentation experiment performed with 2 mg/ml polymerized tubulins and different Ana1 fragments. The experiment was repeated three times with similar results. **(C and D)** Cartoon representations for AF3-predicted Ana1^180–497^ or Ana1^1404–1729^ structural models showing the distribution of surface-exposed positive charges. Residues subjected to mutation are highlighted in the zoomed-in view boxes. **(E)** Confocal images of *in vitro* Taxol-stabilized MTs at 1 μM (ratio of rhodamine-labeled tubulin/unlabeled tubulin is ∼10%; red) mixed with 1 μM Ana1^180–497^ or 2 μM Ana1^1404–1729^ protein. Scale bar, 20 µm. Identical imaging settings were used for all groups. Note that an inset is included for tubulin-only control, showing images of rhodamine-labeled tubulin with different contrasts to the protein–tubulin mixture group for better visualization. Source data are available for this figure: SourceData F7.

**Figure S4.**
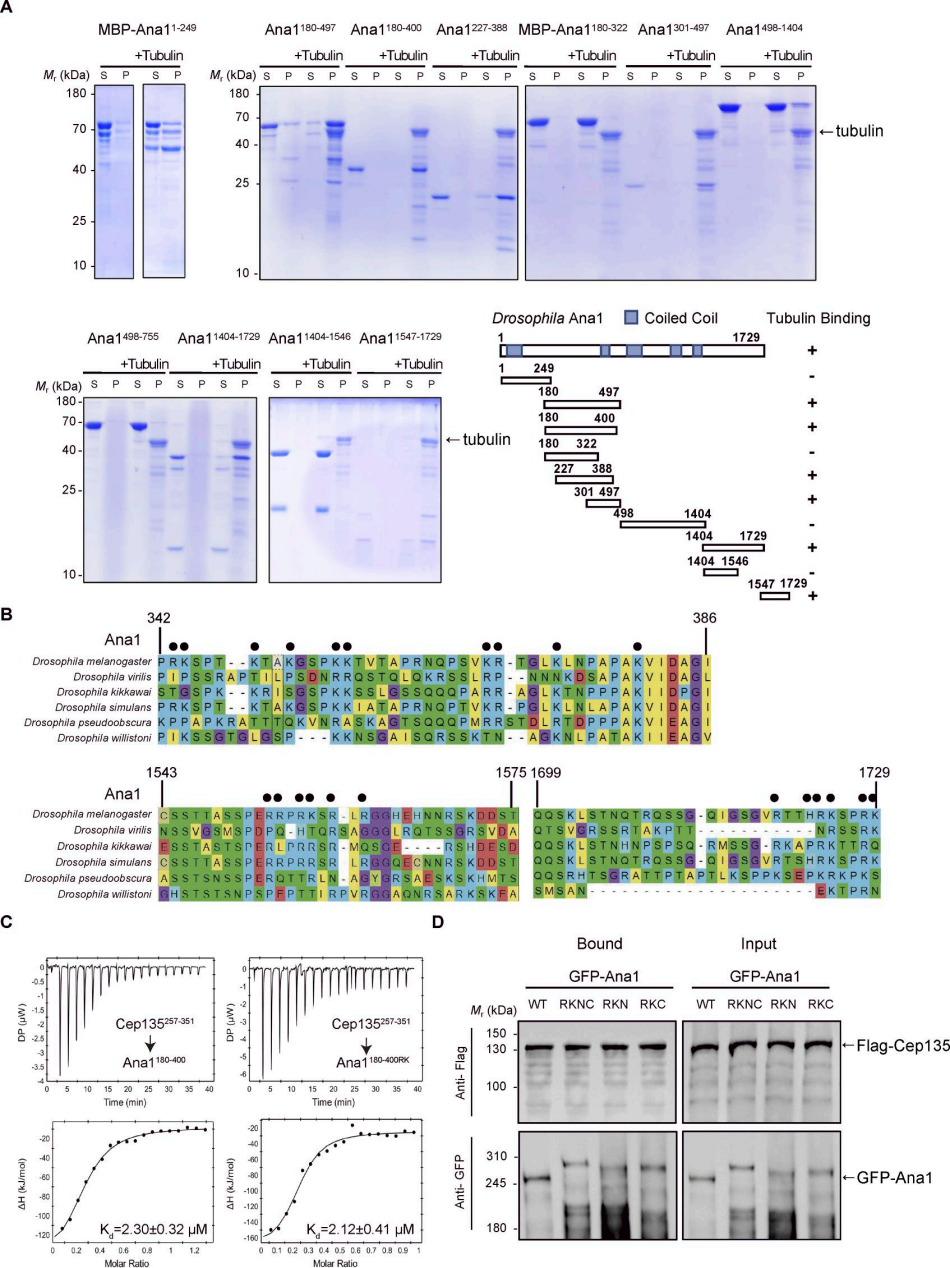
**Mapping of the Ana1-MT interaction region. (A)** SDS-PAGE analysis showing the region of Ana1 that directly interacts with MTs. A schematic representation of the various Ana1 constructs analyzed for binding is shown. **(B)** MSA showing the conservation of Ana1 residues involved in MT binding in *Drosophila* species. **(C)** ITC experiments showing interactions between Cep135^257–351^ and WT Ana1 or MT binding–deficient mutant (Ana1^RKN^). Mutations within the Ana1-MT–binding interface do not affect its interaction with Cep135. **(D)** S2 cells were transiently cotransfected with GFP-tagged Ana1 and Flag-tagged Cep135, along with various MT-binding mutants of Ana1 (RKNC, RKN, and RKC). Cell extracts were subjected to pull-down assays using GFP-Trap beads, and bound proteins were analyzed by western blotting with anti-Flag to detect Cep135. All three Ana1 mutants (RKNC, RKN, and RKC) retained the ability to bind Cep135. The experiment was repeated three times with similar results. WT, wild type; MSA, multiple sequence alignment. Source data are available for this figure: SourceData FS4.

Since the full-length Ana1 protein could not be purified using a recombinant expression system, we instead purified Ana1 fragments and evaluated their ability to bind MTs using cosedimentation assays. We found two regions of Ana1, aa180–497 (hereafter Ana1 N fragment) and aa1404–1729 (hereafter Ana1 C fragment), efficiently coprecipitated with prepolymerized tubulin, indicating direct MT binding (Fig. 7, A and B; and Fig. S4 A). Fluorescence imaging further showed that both fragments could bundle MTs *in vitro*. In the presence of either fragment, the originally short and thin single MT filaments assembled into long, thick fascicles (Fig. 7 E).

To further characterize how Ana1 binds MTs, we analyzed AF3-predicted structural models of the N and C fragments. Surface charge analysis revealed one cluster of positively charged residues in the N fragment and two in the C fragment that are likely exposed for MT binding (Fig. 7, C and D; and Fig. S3 B). These charged patches are highly conserved among all *Drosophila* species (Fig. S4 B). To test their functional importance, we substituted these residues with Ala (referred to as the “RK” mutation). In both fragments, the RK mutations strongly impaired MT binding and bundling, as shown by cosedimentation and fluorescence imaging–based assays, respectively (Fig. 7, A, B, and E). Importantly, ITC-based experiments and GFP pulldowns both confirmed that these MT binding–deficient mutations had no influence on the Ana1-Cep135 interaction (Fig. S4, C and D).

### The Ana1-MT interaction promotes centriole elongation *in vivo*

**Figure 8.**
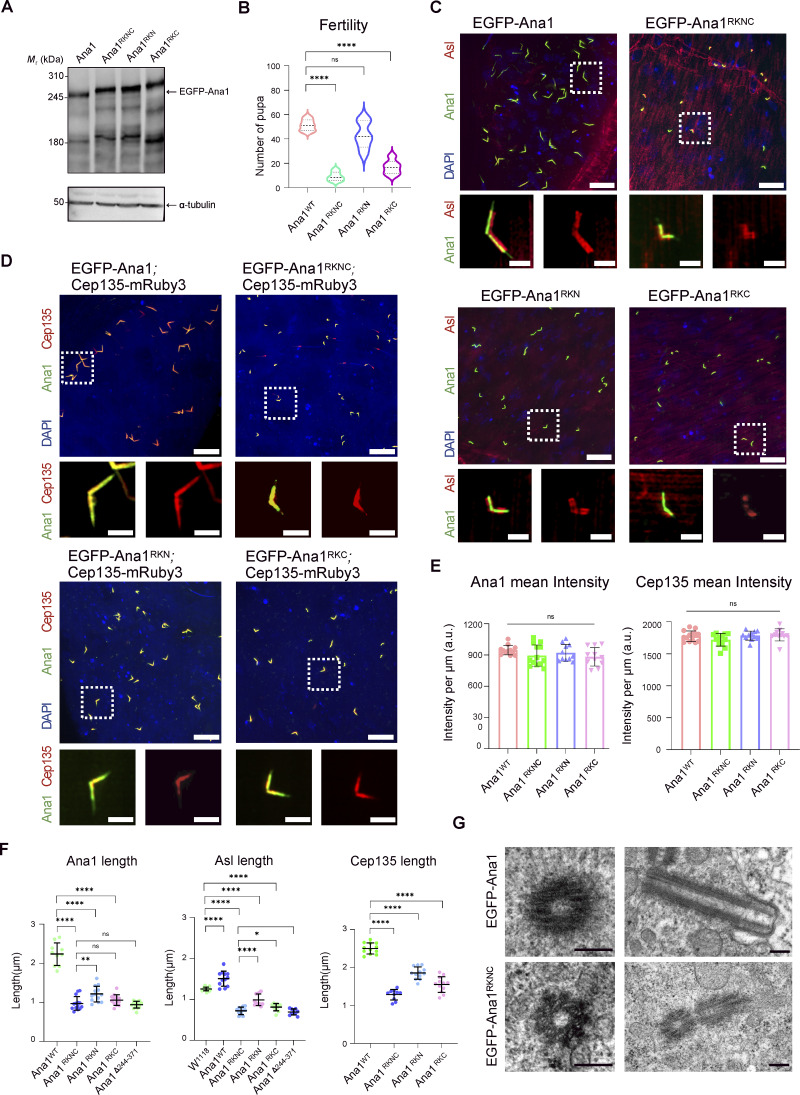
**Ana1-MT interaction is essential for Ana1-mediated centriole elongation in primary spermatocytes. (A)** Western blot of extracts from 50 to 60 testes for indicated genotypes. Immunoblot analysis of Ana1 protein variants with an anti-Ana1 primary antibody. α-Tubulin is shown as a loading control. **(B)** Graphs showing the male fertility of different genotypes, as measured by the number of progeny pupae produced from mating with WT (*w*^*1118*^) virgin females. ****P < 0.0001. ns, not significant. **(C and D)** Centrioles in primary spermatocytes stained to reveal EGFP-Ana1 (green), Asl (red), Cep135-mRuby3 (red), and DNA (blue). Scale bar, 10 µm (overview image) or 2 µm (inset image). **(E)** Graphs showing the mean ± SD quantification for Ana1 and Cep135 mean intensity per unit length of centrioles in primary spermatocytes. Each point on the graph represents the average mean intensity measured from a 16-cell cyst with 10–15 centrioles scored; five independent testes were measured for each genotype with 2–3 cell cysts scored in each testis. ns, not significant. **(F)** Graphs showing the mean ± SD quantification for centriole lengths in mature primary spermatocytes using Ana1, Asl, or Cep135 as a centriole marker for each genotype. Each point on the graphs represents the average length measured from a 16-cell cyst with 10–15 centrioles scored; five independent testes were measured for each genotype with 2–3 cell cysts scored in each testis. *P < 0.05, **P < 0.01, ****P < 0.0001. ns, not significant. **(G)** Cross-sections (left) of centrioles in *Drosophila* primary spermatocytes by TEM showing preserved ninefold symmetry in both EGFP-Ana1 (top) and EGFP-Ana1-RKNC (bottom) groups. Longitudinal sections (right) show that the RKNC mutation leads to centriole shortening compared with full-length Ana1 controls. For each genotype, centrioles were examined from at least three independent testes, with a total of 8–12 centrioles analyzed. Scale bar, 200 nm. WT, wild type. Source data are available for this figure: SourceData F8.

To test whether the MT-binding activity of Ana1 is essential for centriole elongation *in vivo*, we introduced RK mutations into EGFP-tagged full-length Ana1 protein to disrupt either the N-terminal site (hereafter referred to as Ana1^RKN^), the C-terminal site (hereafter referred to as Ana1^RKC^), or both (hereafter referred to as Ana1^RKNC^). All variants were expressed under a ubiquitin promoter and could fully rescue the uncoordination phenotype of *ana1* mutant flies (Fig. 8 A and Fig. S2 C). While male fly fertility was partially restored in all genotypes, only Ana1^RKN^ supported pupal production at near-WT levels (Fig. 8 B).

**Figure S5.**
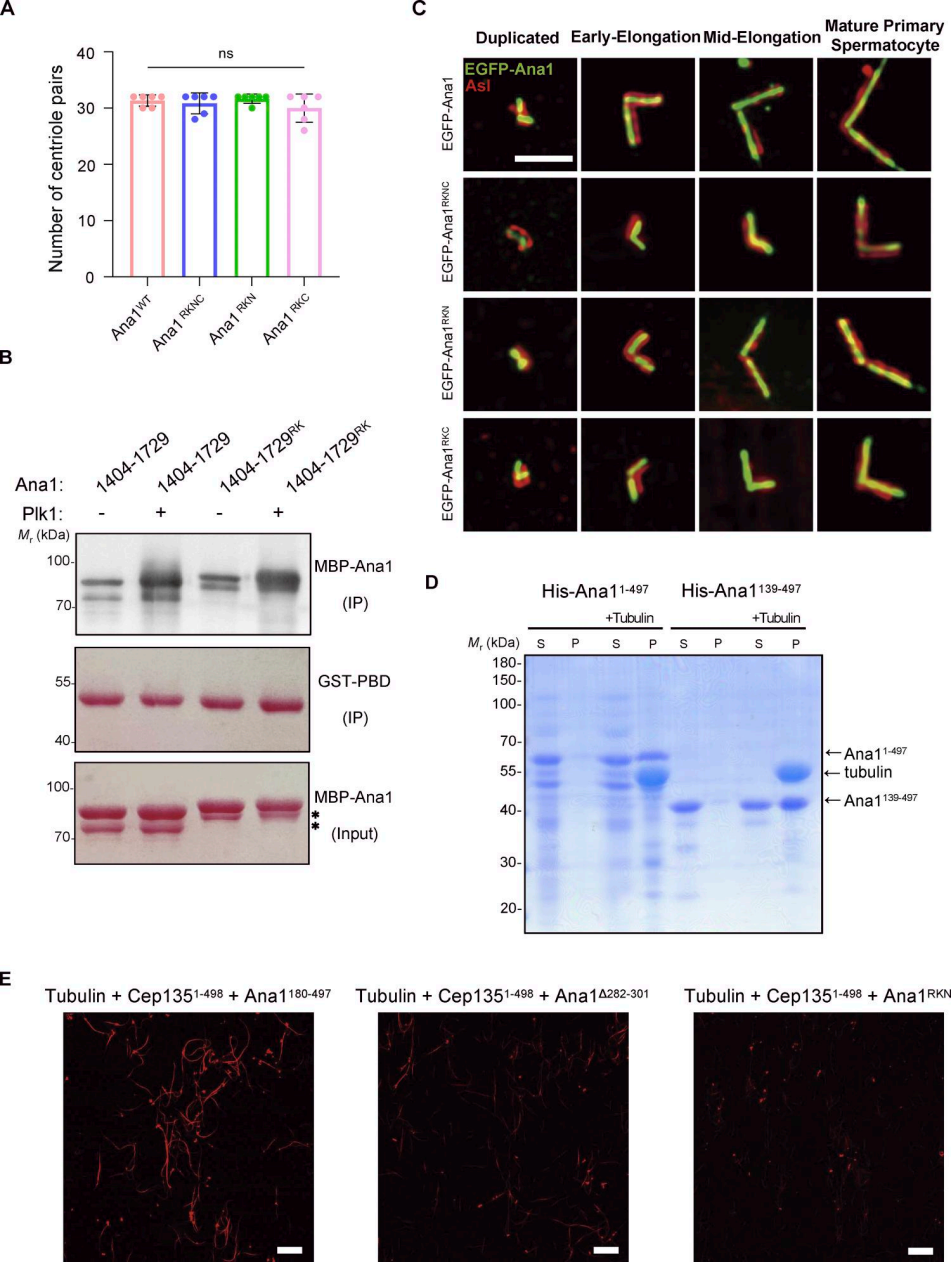
**MT binding–deficient Ana1 mutants fail to induce centriole overelongation. (A)** Representative images showing centriole elongation in primary spermatocytes at various stages from different genotypes, stained for EGFP-Ana1 (green) and Asl (red). Scale bar, 2 µm. **(B)** Centriole pairs, marked by Asl localization, were quantified in 15–20 cells per testis from the indicated genotypes. Each data point represents the number of centriole pairs per testis, derived from five independent testes per genotype. Error bars indicate the mean ± SD. ns, not significant (unpaired two-tailed Student’s *t* test). **(C)***In vitro* GST pull-down assay showing interactions between MBP-Ana1^1404–1729^ or Ana1^1404–1729RK^ and GST-PBD when incubated with or without PLK (indicated as “Plk1 +/−”). Top panel, western blot analysis of MBP-Ana1 being pulled down by GST-PBD. Note that the binding was enhanced by PLK1 phosphorylation and the RK mutation had no effect on protein binding. Middle panel, SDS-PAGE analysis showing comparable amount of GST-PBD coupled to the GST beads. Bottom panel, SDS-PAGE analysis showing MBP-Ana1 input. Asterisks highlight the degradation band of MBP-Ana1. **(D)** Coomassie blue–stained SDS-PAGE gel of a MT cosedimentation experiment performed with 2 mg/ml polymerized tubulins and His-Ana1^1–497^ or His-Ana1^139–497^(CR1 deletion). **(E)** Rhodamine-labeled tubulin (red; ∼10% labeling ratio with unlabeled tubulin) was mixed with 1 μM Taxol-stabilized MTs in combination with 1 μM Cep135^1–498^, 1 μM Ana1^180–497^, 1 μM Ana1^Δ282–301^, or 1 μM Ana1^RKN^ as indicated. Identical imaging settings were used across all groups for direct comparison. Scale bar, 20 μm. PBD, Polo-box domain. Source data are available for this figure: SourceData FS5.

Using Asl and Cep135 as centriole markers, we found that MT binding–deficient Ana1 mutants strongly localized to centrioles and centriole duplication was not affected (Fig. 8 C and Fig. S5 B). However, these mutants failed to promote centriole overelongation to the same extent as WT Ana1 (Fig. 8, C and D; and Fig. S5 A). Further quantification revealed that the amounts of Ana1 and Cep135 recruited per unit length of centrioles were not significantly altered (Fig. 8 E), and therefore, the elongation defect was not likely due to impaired centriolar recruitment. The severity of centriole shortening followed a gradient: Ana1^RKN^ showed the mildest effect, Ana1^RKC^ a stronger reduction, and Ana1^RKNC^ the most severe defect (Fig. 8 F). Furthermore, we compared the length of centrioles in spermatocytes expressing MT binding–deficient (Ana1^RKNC^) or Cep135 binding–deficient (Ana1^Δ244–371^) mutants, and found that the two types of mutations showed a comparable reduction in centriole length as measured by Asl (Fig. 8 F). And in either case, no obvious structural defects were detected, as confirmed by TEM (Fig. 8 G).

A recent study reported that Ana1 mutants deficient in Polo kinase binding also showed defects in centriole elongation (Alvarez-Rodrigo et al., 2021). To rule out the possibility that elongation defects observed in RK mutants were due to impaired Polo kinase interaction, we conducted GST pull-down experiments. It was demonstrated that the Ana1^1404–1729RK^ mutant protein retained full binding to the Polo-box domain (Fig. S5 C). We also examined whether the conserved CR1 region of Ana1, whose deletion had no detectable effect on Cep135 binding, might be involved in MT association. The CR1-deleted Ana1 protein could still cosediment with prepolymerized tubulin (Fig. S5 D).

Taken together, these results indicate that the MT-binding activity of Ana1 is required for centriole overelongation *in vivo* and that disruption of this activity leads to defects in elongation that are comparable in severity to those caused by disruption of the Ana1-Cep135 interaction.

## Discussion

Ana1/CEP295 proteins are crucial for centriole-to-centrosome conversion and have been implicated in centriole elongation, but the mechanisms through which they drive giant centriole assembly during spermatogenesis remain poorly understood. Our study demonstrates that Ana1 promotes centriole elongation through two molecular activities—binding to Cep135 and direct association with MTs. Disruption of either interaction suppressed Ana1-induced centriole overelongation and reduced centriole lengths below the endogenous level. Intriguingly, both types of mutants converged on comparable centriole lengths, with ∼0.7227 μm for Ana1^Δ244–371^ centrioles and 0.6958 μm for Ana1^RKNC^ centrioles as measured by the Asl signal. This observation led us to propose that there might be a shared structural limit imposed by the proximal centriolar scaffold, such as the cartwheel, the A-C linker, and/or the inner scaffold, which has been demonstrated to reinforce the MT wall (Cai et al., 2025; Guichard et al., 2013; Klena et al., 2020; Le Guennec et al., 2020; Li et al., 2019; Nazarov et al., 2020; Ruehle et al., 2024). Beyond this proximal core, continued longitudinal extension of the outer MT triplets likely requires additional scaffolding provided by the Cep135-Ana1 complex and by Ana1’s direct MT-binding activity. Flies expressing these binding-deficient Ana1 mutants exhibited no coordination defects, which suggests that neurosensory cilia remained functionally intact. Their male fertility was nonetheless only partially rescued, which underscores the importance of correct centriole lengths for gametogenesis.

We mapped MT binding to positively charged patches within the Ana1^1404–1729^ region and showed that these residues are required for centriole elongation. In line with this, a recent study reported that Ana1^1472–1620^ is required for centriole elongation, although the underlying mechanism was not elucidated (Nagy et al., 2025). Notably, deletion of this region completely abolished Ana1’s ability to rescue male fertility in *ana1*^*null*^ flies, whereas our point mutants within the same region only partially impaired male fertility. This phenotypic difference suggests that the Ana1^1472–1620^ region might mediate additional interactions beyond MT binding that also contribute to centriole function during spermatogenesis. In addition, Ana1 also binds MTs through its N-terminal region, although this contribution appears weaker. We speculate that these dual MT-binding interfaces may facilitate the longitudinal stacking of Ana1 and its associated complexes, thereby promoting the elongation of MT triplets during the formation of sperm-specific giant centrioles. In relation to this, human Cep295 binds centriolar MTs at the proximal end and stabilizes the MT wall independently of Cep135 (Atorino et al., 2020). It also promotes the assembly of the distal half of centrioles through recruitment of POC5 and POC1B, which then triggers posttranslational modification of centriolar MTs (Chang et al., 2016). Together, these findings suggest a conserved role of Ana1/CEP295 in direct MT binding and centriole elongation.

Cep135 has also been reported to bind MTs *in vitro* and in cultured cells (Carvalho-Santos et al., 2012). It is therefore plausible that Ana1, Cep135, and MTs stabilize one another within a ternary complex that reinforces the centriolar MT wall and supports continued longitudinal extension of the outer MT triplets. In support of this model, our *in vitro* reconstitution assay showed that a mixture of the Ana1 N fragment with MBP/Cep135^1–498^ promoted the formation of meshwork-like MT assemblies; disruption of Ana1’s interaction with either Cep135 or MTs, however, dramatically reduced the formation of such assemblies (Fig. S5 E). Structural characterization of these molecular scaffolds will be important to fully understand how Ana1 mediates centriole elongation beyond the cartwheel-containing core. In addition, how impaired centriole elongation propagates through axoneme assembly to ultimately affect male fertility will be another important direction for future investigation.

Our data suggest that Cep135 does not promote centriole elongation by simply recruiting Ana1 to centrioles. Cep135 binding–deficient Ana1 mutants still localized robustly to centrioles in a homozygous *ana1* null background. This is consistent with earlier observations in fly embryos, where the expression of the Ana1 C-terminal region (lacking the N-terminal Cep135-binding domain) was sufficient for weak centrosomal localization and could partially rescue centrosome assembly defects in *ana1*^*mecB*^ mutant flies (Saurya et al., 2016). However, interpretation of that study was limited by the presence of the endogenous Ana1^1–1120^ protein, which might facilitate its recruitment via self-interaction (Nagy et al., 2025). To overcome this, we utilized a new *ana1* null mutant allele that was created using CRISPR/Cas9 to delete the entire coding region and thereby confirmed Cep135-independent Ana1 recruitment in primary spermatocytes. This contrasts with findings in cultured D.Mel-2 cells, where Cep135 acts upstream of Ana1 and is required for its centriolar localization (Fu et al., 2016). Rcd4 has also been implicated in Ana1 recruitment in D.Mel-2 cells (Panda et al., 2020), yet Ana1 localization appeared unperturbed in *rcd4* null mutants during spermatogenesis (Panda et al., 2024). These results altogether indicate that the precise mechanism of Ana1 recruitment may vary between cell types and may involve redundant or alternative pathways. Future studies are clearly required to identify the full repertoire of proteins involved in Ana1 recruitment and to determine how these pathways are regulated across different developmental stages and in different cell types.

Although Cep135 is dispensable for Ana1 recruitment in primary spermatocytes, the dependency between the two proteins is reversed at later stages of spermatogenesis. We found that the Ana1-Cep135 interaction is required for loading Cep135 into the PCL structure during sperm individualization. Since Ana1 is indeed recruited to the PCL structure before Cep135 (Blachon et al., 2009), this reversed dependency highlights a context-dependent pathway for Cep135 recruitment that is specific to spermiogenesis.

Taken together, our findings reveal that Ana1 promotes centriole elongation through two separable but complementary binding activities—via Cep135 and MTs. These associations appear to become particularly important as the centriole extends beyond what its proximal scaffold alone can sustain. How Ana1-containing complexes assemble and reorganize as centrioles elongate, and whether the same mechanisms underlie Ana1 function in somatic cell types, will be important questions for future structural and mechanistic studies.

## Materials and methods

### Fly stocks

Flies were kept at 25°C on standard *Drosophila* culture medium. The *ana1* null mutant flies were kindly gifted from the Jordan Raff lab, University of Oxford, Oxford, UK. To generate the CRISPR/Cas9-mediated *ana1* knockout allele, two gRNAs (one for each end of the *ana1* coding region; gRNA sequence #1: 5′-GCTCTGCAGCTAACAGTAAA[TGG]-3′ and gRNA sequence #2: 5′-TCCTCAGGTCAAATAGGATC[AGG]-3′) were cloned into the pCFD4 (U6:1-gRNA U6:3-gRNA) plasmid (RRID: Addgene_49411) (Port et al., 2014; Port et al., 2015) using primers with the following sequences:

*Forward primer:* 5′-GCG​GCC​CGG​GTT​CGA​TTC​CCG​GCC​GAT​GCA​GCT​CTG​CAG​CTA​ACA​GTA​AAG​TTT​TAG​AGC​TAG​AAA​TAG​CAA​G-3′
*Reverse primer:* 5′-ATT​TTA​ACT​TGC​TAT​TTC​TAG​CTC​TAA​AAC​GAT​CCT​ATT​TGA​CCT​GAG​GAT​GCA​CCA​GCC​GGG​AAT​CGA​ACC​C-3′.

The resulting plasmids were injected into BL25709 flies (*y, v, nos-int; attp40*) (RRID: BDSC_25709) by the University of Cambridge Department of Genetics Fly Facility to generate gRNA-transgenic flies through attP-mediated mutagenesis. These transgenic flies were then crossed with the previously described Cas9-expressing fly line BL54591 (RRID: BDSC_54591) (Port et al., 2014). The *ana1 null allele* was isolated from a single founder male from the second-generation progeny, and the entire gene locus was subsequently sequenced to confirm the deletion. Two independent null alleles were obtained (Fig. 1 A), denoted as *ana1*^*Δa*^ and *ana1*^*Δb*^, which were subsequently crossed to generate the *ana1* null mutant background used in this study (referred to as *ana1*^*−/−*^).

The following fly stocks were used in this study: *w*^*1118*^ (used as a WT control; RRID: BDSC_3605), *ana1*^*−/−*^*, cep135*^*c04199*^ (Mottier-Pavie and Megraw, 2009), pUbq-RFP-Sas6 (gift from the Jordan Raff lab, University of Oxford), pUbq-RFP-Rcd4 (gift from the Jordan Raff lab, University of Oxford), pUbq-Cep135-mRuby3, pUbq-EGFP-Ana1, pUbq-EGFP-Ana1^Δ244–371^, pUbq-EGFP-Ana1^F283D^, pUbq-EGFP-Ana1^I286D^, pUbq-EGFP-Ana1^RKNC^, pUbq-EGFP-Ana1^RKN^, pUbq-EGFP-Ana1^RKC^, and pUbq-EGFP-Ana1^Δ25–138^.

Full-length Ana1 constructs used in this study were made by cloning the full-length Ana1 cDNA into the pUbq-EGFP vector using homologous recombination (Vazyme). Single-point mutations and truncation mutations were introduced into the full-length Ana1 using overlapping PCR (Vazyme). All constructs were injected by the Core Facility of *Drosophila* Resource and Technology (CEMCS) via random P-element insertion into a *w*^*1118*^ background.

### Fertility assay

One male fly 3 days after eclosion was crossed with five *w*^*1118*^ virgin flies and then transferred into a new tube after 2 days. 10 days later, the number of pupae on the wall of the tube was counted. 15 groups of experiments were conducted for each genotype and repeated three times.

### *Drosophila* protein-level analysis and antibody generation

Testes from ∼50–60 adult males per genotype were dissected in PBS, homogenized in 50 μl of 1% RIPA lysis buffer (P0013B; Beyotime Biotechnology), and subjected to overnight lysis at 4°C. Following centrifugation at 13684 × *g* for 10 min, the supernatant was collected and combined with 5× loading dye. Proteins were separated by SDS-PAGE, transferred onto nitrocellulose membranes (0.22 μm; E804-01; Vazyme), and subjected to western blotting analysis using rabbit polyclonal anti-Ana1 antibody (1:2,000, this study) and rabbit polyclonal anti-Cep135 antibody (1:2,000, this study) to detect target proteins.

Polyclonal antibodies against Ana1 and Cep135 were generated by immunizing New Zealand White rabbits with purified protein fragments (Ana1: aa 1404–1729; Cep135: aa 1–204), and the final antibodies were affinity-purified from the serum. All animal immunization and antibody purification procedures were performed by UNoK Bio (Suzhou).

### Immunofluorescence and spinning disk confocal microscopy

Testes from adult male flies were dissected in PBS and fixed in 4% PFA for 30 min. Then, testes were subjected to three quick, 5-min washes in PBS containing 0.2% Triton X-100, followed by blocking with 10% NGS for 1 h, and incubated in primary antibodies at 4°C overnight. Testes were then washed with PBS three times and incubated with the secondary antibody for 1 h at room temperature. Finally, testes were washed again with PBS three times and mounted with Antifade Mounting Medium with DAPI (Beyotime). The following antibodies were used: rabbit polyclonal anti-Asl antibody (1:250; this study), rabbit polyclonal anti-dPLP antibody (1:200; this study), goat anti-rabbit IgG (H+L) Alexa Fluor 594–conjugated antibody (1:400; 33112ES60; Yeason; RRID: AB_3661961).

All the slices were examined by the Olympus SpinSR10 spinning disk confocal microscope system using UPLAPO100XOHR (100×/1.50 NA, oil immersion, WD 0.12 mm) objective with a correction collar, at 23°C, and a Hamamatsu ORCA-Flash4.0 sCMOS camera (model C13440-20CU-USB3.0) in a SR mode. Images were processed and analyzed with ImageJ (ImageJ/Fiji, NIH; RRID: SCR_002285).

### Centriole length and fluorescence intensity measurements

In order to avoid ambiguity contributed by tilting, only centrioles oriented perpendicular to the imaging axis were measured. The entire centriole volume was scanned using system-optimized *z-*stack steps on the Olympus SpinSR10 spinning disk confocal microscope system as described above. The length of the centrioles was measured using the line profile tool in ImageJ (ImageJ/Fiji, NIH) and statistically analyzed. Quantification of Ana1 fluorescence intensity and mean intensity per μm of centriole length in spermatocytes was performed in Fiji using the line tool (line width = 9). Background fluorescence was subtracted before obtaining the final value.

### Negative geotaxis assay

3-day-old adult flies of each genotype were collected and placed into empty polystyrene culture vials (15 flies per vial) and allowed to acclimate for 15–20 min at room temperature. To initiate the assay, the vial was firmly tapped three times against the benchtop to knock all flies to the bottom. The number of flies that climbed above a 5-cm-height mark within the recording period was counted, and the percentage of successful climbers was calculated. Three independent biological replicates were performed per genotype, and statistical significance relative to the control was determined using unpaired two-tailed Student’s *t* test.

### TEM of *Drosophila* testes

Testes were rapidly dissected from adult flies in ice-cold 1× PBS and immediately fixed in 2.5% glutaraldehyde (in 0.1 M phosphate buffer, pH 7.4) at 4°C for at least 4 h. Following fixation, samples were washed three times (15 min each) in 0.1 M phosphate buffer and postfixed in 1% osmium tetroxide for 1.5 h. After three additional buffer washes (15 min each), specimens were dehydrated through a graded acetone series: 50%, 70%, and 90% acetone (15 min each), followed by three changes of 100% acetone (15–20 min each). Samples were infiltrated and embedded in Epon812 resin as follows: pure acetone/resin mixture (2:1) for 0.5 h at room temperature, pure acetone/resin mixture (1:2) for 1.5 h at 37°C, and pure resin for 2 h at 37°C. Polymerization was performed sequentially at 37, 45, and 60°C for 24 h at each temperature. Ultrathin sections (70 nm) were cut using a Reichert-Jung ULTRACUT E ultramicrotome, stained with uranyl acetate for 15 min and lead citrate for 10 min, and examined with a JEOL JEM-1400F transmission electron microscope operated at an appropriate accelerating voltage. To analyze centriole ultrastructure in primary spermatocytes, longitudinal sections along the centriole axis and cross-sections perpendicular to the centriole axis were acquired at 20,000× magnification.

### Statistical analysis

The population size and sample size are indicated in corresponding figures and figure legends. Each sample has been randomly selected from the populations from which the samples are derived. Data analysis was carried out by GraphPad Prism (version 9.0.0; RRID: SCR_002798), and the data were presented as the mean ± SD. Statistical significances were analyzed using unpaired two-tailed Student’s *t* test when the assumptions of normal data distribution and equal population variance were satisfied. The significance was established as a *P* value of ≤0.05.

### Cell culture and co-immunoprecipitation

S2 cells (RRID: CVCL_Z232) were cultured in Schneider’s *Drosophila* Medium (Gibco, [+] L-glutamine, LOT: 2872618) with 10% inactivated FBS (Gibco, LOT: 2707023RP). Transfection of plasmids was performed using Transfection Reagent (Cat. No. 301427; QIAGEN). 24 h after passaging, cells in each dish were cotransfected with 1 µg pAWF and 1 µg pAWG-tagged plasmids (RRID: DGRC_1072). The medium was replaced 24 h after transfection. Cells were harvested 72 h after transfection, lysed with lysis buffer (0.5% NP-40 in TBS buffer, Beyotime Biotechnology) for 1 h at 4°C, and centrifuged to collect the supernatant. The GFP beads (Cat. No. SA070005; Smart-Lifesciences, Lot No. A24011601) (soaked in lysis buffer for 1 h in advance) were combined with the supernatant at 4°C for 2 h and washed with 1 ml washing buffer (lysis buffer diluted five times) three times. Finally, SDS-PAGE and western blotting (mouse monoclonal anti-Flag antibody [1;2,000, Cat. No. F1804; Sigma-Aldrich; RRID: AB_262044] and rabbit anti-GFP [1:2,000, ab290; Abcam; RRID: AB_2313768]) were performed to test the binding of the proteins.

### Plasmid construction and protein expression purification

The cDNA sequence encoding full-length Ana1 or Cep135 was derived from *Drosophila* embryos. DNA encoding various fragments or mutants was cloned into a pET-32m3C or pET-MBP3C vector (RRID: Addgene_109029) to create an ORF with an N-terminal His6 tag or MBP-His6 tag, respectively. Proteins were expressed in *Escherichia coli* BL21 (DE3) strains in LB broth at 16°C, and purified using Ni-NTA chromatography followed by size-exclusion chromatography (50 mM Tris, pH 7.8, 300 mM NaCl, 1 mM DTT). The N-terminal His or MBP-His tag was cleaved off using His-3C protease, and the untagged protein was further purified via size-exclusion chromatography (50 mM Tris, pH 8.0, 300 mM NaCl, 1 mM DTT).

### Isothermal titration calorimetry

ITC experiments were conducted using a MicroCal PEAQ-ITC (Malvern Panalytical) system. All proteins were prepared in the same reaction buffer containing 20 mM Tris, pH 8.0, 150 mM NaCl, and 1 mM DTT. The protein loaded in the syringe was highly concentrated (400 μM) for titrating into its binder in the reaction cell (40 μM). Reaction was performed at 25°C. Each titration point injected 2-μl syringe protein into the cell within 4 s, following by 120-s equilibrium. A titration curve contained a total of 18 titration points. Each titration curve was fitted with the one-site binding model using Malvern ITC Analysis Software to obtain K_d_ and binding stoichiometry (N).

### Tubulin polymerization with Taxol

Lyophilized tubulin powders (Cat. No. T240; Cytoskeleton) were dissolved in PEM buffer (80 mM PIPES, 1 mM EGTA, and 1 mM MgCl_2_, pH 6.8) and incubated on ice for 5 min to prepare a 20 mg/ml tubulin stock solution. The stock solution was then diluted to a final concentration of 2 mg/ml with PEM buffer containing 1 mM GTP (Sigma-Aldrich) and 1 mM DTT (Sigma-Aldrich) to form a polymerization mixture. For confocal imaging, 1 μl of rhodamine-labeled tubulin (Cat. No. TL590M; Cytoskeleton) was added to the tubulin preparation. The mixture was centrifuged at 150,000 × *g* for 5 min at 4°C to remove small aggregates. The supernatant was incubated at 37°C for 1 h. Taxol (paclitaxel, Absin) was added stepwise to the polymerization mixture, starting from 2 μM and increasing up to 200 μM, with 10-min incubations at 37°C between each increment to allow for full polymerization.

### MT cosedimentation with recombinant proteins

A 50-μl aliquot of the polymerized MTs was mixed with 100 μl recombinant protein (at concentrations of 10 μM) in reaction buffer (10 mM HEPES, pH 7.7, 50 mM KCl, 1 mM DTT, 20 μM Taxol). The recombinant proteins were centrifuged at 150,000 × *g* for 5 min to remove any potential aggregates prior to use. The protein and MT mixture were incubated at room temperature for 15 min. It was then gently added into a centrifuge tube prefilled with 150 μl of sucrose cushion solution (10 mM HEPES, pH 7.7, 50 mM KCl, 40% wt/vol sucrose, 20 μΜ Taxol). The mixture was centrifuged at 150,000 × *g* for 20 min at 25°C.

After centrifugation, 100 μl of the supernatant was carefully withdrawn, while the rest was discarded. The pellet fraction was washed twice with reaction buffer. Both the supernatant and pellet fractions were resuspended in 100 μl SDS-PAGE loading buffer (125 mM Tris-HCl, pH 6.8, 10% SDS, 20% glycerol, 10% β-mercaptoethanol). The samples were boiled at 95°C to dissolve the pellets and analyzed by SDS-PAGE followed by Coomassie blue staining.

### Imaging of tubulin polymerization

The reaction mixture containing 10% rhodamine-labeled tubulin (Cytoskeleton) at 1 μm and specified concentrations of protein components was prepared in reaction buffer (50 mM Tris-HCl, pH 8.0, 100 mM KCl, 1 mM DTT, 20 μM Taxol). The mixture was loaded into a custom-designed imaging chamber and incubated at room temperature for 20 min to facilitate polymerization, which was then subjected to fluorescence imaging at room temperature using a Zeiss LSM 880 confocal microscope equipped with two MA-PMT and a GaAsP-PMT detectors; with a 63×/1.40 NA oil objective lens. Images were acquired and processed using ImageJ (ImageJ/Fiji, NIH).

### Circular dichroism

The protein was purified and diluted in phosphate buffer (0.1–1 mg/ml), and the circular dichroism spectrometer (Applied Photophysics Ltd., Chirascan) was pretreated for 30 min. Baseline scans were performed with a quartz cuvette (0.1 cm path length). Then, the sample was loaded avoiding bubbles, scanned from 190 to 260 nm, and repeated three times with the results averaged. The data were analyzed using DichroWeb to determine secondary structures (e.g., α-helix, β-sheet). Minima at 208 and 222 nm indicate α-helical content.

### Online supplemental material

Fig. S1 shows mapping of the interaction region between Cep135 and Ana1. Fig. S2 shows Cep135 binding–deficient Ana1 mutants fail to induce centriole overelongation. Fig. S3 shows Predicted Aligned Error plots for AF3-predicted structures. Fig. S4 shows mapping of the Ana1-MT interaction region. Fig. S5 shows MT binding–deficient Ana1 mutants fail to induce centriole overelongation.

## Supplementary Material

SourceData F1 is the source file for Fig. 1.

SourceData F2 is the source file for Fig. 2.

SourceData F3 is the source file for Fig. 3.

SourceData F4 is the source file for Fig. 4.

SourceData F5 is the source file for Fig. 5.

SourceData F7 is the source file for Fig. 7.

SourceData F8 is the source file for Fig. 8.

SourceData FS1 is the source file for Fig. S1.

SourceData FS4 is the source file for Fig. S4.

SourceData FS5 is the source file for Fig. S5.

## Data Availability

All data supporting the findings of this study are available within the paper and its supplemental materials, including source data files. Additional data are available from the corresponding author upon reasonable request.

## References

[bib1] Abramson, J., J. Adler, J. Dunger, R. Evans, T. Green, A. Pritzel, O. Ronneberger, L. Willmore, A.J. Ballard, J. Bambrick, . 2024. Accurate structure prediction of biomolecular interactions with AlphaFold 3. Nature. 630:493–500. 10.1038/s41586-024-07487-w 38718835 PMC11168924

[bib2] Alvarez-Rodrigo, I., A. Wainman, S. Saurya, and J.W. Raff. 2021. Ana1 helps recruit Polo to centrioles to promote mitotic PCM assembly and centriole elongation. J. Cell Sci. 134:jcs258987. 10.1242/jcs.258987 34156068 PMC8325959

[bib3] Arquint, C., A.M. Gabryjonczyk, S. Imseng, R. Bohm, E. Sauer, S. Hiller, E.A. Nigg, and T. Maier. 2015. STIL binding to Polo-box 3 of PLK4 regulates centriole duplication. Elife. 4:e07888. 10.7554/eLife.07888 26188084 PMC4530586

[bib4] Atorino, E.S., S. Hata, C. Funaya, A. Neuner, and E. Schiebel. 2020. CEP44 ensures the formation of bona fide centriole wall, a requirement for the centriole-to-centrosome conversion. Nat. Commun. 11:903. 10.1038/s41467-020-14767-2 32060285 PMC7021698

[bib5] Azimzadeh, J. 2014. Exploring the evolutionary history of centrosomes. Philos. Trans. R. Soc. Lond. B Biol. Sci. 369:20130453. 10.1098/rstb.2013.0453 25047607 PMC4113097

[bib6] Banterle, N., A.P. Nievergelt, S. de Buhr, G.N. Hatzopoulos, C. Brillard, S. Andany, T. Hubscher, F.A. Sorgenfrei, U.S. Schwarz, F. Grater, . 2021. Kinetic and structural roles for the surface in guiding SAS-6 self-assembly to direct centriole architecture. Nat. Commun. 12:6180. 10.1038/s41467-021-26329-1 34702818 PMC8548535

[bib7] Bettencourt-Dias, M., F. Hildebrandt, D. Pellman, G. Woods, and S.A. Godinho. 2011. Centrosomes and cilia in human disease. Trends Genet. 27:307–315. 10.1016/j.tig.2011.05.004 21680046 PMC3144269

[bib8] Bettencourt-Dias, M., A. Rodrigues-Martins, L. Carpenter, M. Riparbelli, L. Lehmann, M.K. Gatt, N. Carmo, F. Balloux, G. Callaini, and D.M. Glover. 2005. SAK/PLK4 is required for centriole duplication and flagella development. Curr. Biol. 15:2199–2207. 10.1016/j.cub.2005.11.042 16326102

[bib9] Blachon, S., X. Cai, K.A. Roberts, K. Yang, A. Polyanovsky, A. Church, and T. Avidor-Reiss. 2009. A proximal centriole-like structure is present in *Drosophila* spermatids and can serve as a model to study centriole duplication. Genetics. 182:133–144. 10.1534/genetics.109.101709 19293139 PMC2674812

[bib11] Buglak, D.B., K.H.M. Holmes, B.J. Galletta, and N.M. Rusan. 2024. The proximal centriole-like structure maintains nucleus-centriole architecture in sperm. J. Cell Sci. 137:jcs262311. 10.1242/jcs.262311 39166297 PMC11423811

[bib12] Cai, B., J. Xu, E.H. Collet, E. Aarts, L. Luo, A. Leitner, T. Ishikawa, P. Beltrao, C.G. Pearson, M. Pilhofer, and M. Wieczorek. 2025. Structure and assembly of the A-C linker connecting microtubule triplets in centrioles. Sci. Adv. 11:eady3689. 10.1126/sciadv.ady3689 41061066 PMC12506977

[bib13] Carvalho-Santos, Z., P. Machado, I. Alvarez-Martins, S.M. Gouveia, S.C. Jana, P. Duarte, T. Amado, P. Branco, M.C. Freitas, S.T. Silva, . 2012. BLD10/CEP135 is a microtubule-associated protein that controls the formation of the flagellum central microtubule pair. Dev. Cell. 23:412–424. 10.1016/j.devcel.2012.06.001 22898782

[bib14] Chang, C.W., W.B. Hsu, J.J. Tsai, C.J. Tang, and T.K. Tang. 2016. CEP295 interacts with microtubules and is required for centriole elongation. J. Cell Sci. 129:2501–2513. 10.1242/jcs.186338 27185865 PMC4958302

[bib15] Conduit, P.T., A. Wainman, and J.W. Raff. 2015. Centrosome function and assembly in animal cells. Nat. Rev. Mol. Cell Biol. 16:611–624. 10.1038/nrm4062 26373263

[bib16] Cottee, M.A., N. Muschalik, Y.L. Wong, C.M. Johnson, S. Johnson, A. Andreeva, K. Oegema, S.M. Lea, J.W. Raff, and M. van Breugel. 2013. Crystal structures of the CPAP/STIL complex reveal its role in centriole assembly and human microcephaly. Elife. 2:e01071. 10.7554/eLife.01071 24052813 PMC3776556

[bib17] Dutcher, S.K. 2003. Elucidation of basal body and centriole functions in *Chlamydomonas reinhardtii*. Traffic. 4:443–451. 10.1034/j.1600-0854.2003.00104.x 12795689

[bib18] Fu, J., Z. Lipinszki, H. Rangone, M. Min, C. Mykura, J. Chao-Chu, S. Schneider, N.S. Dzhindzhev, M. Gottardo, M.G. Riparbelli, . 2016. Conserved molecular interactions in centriole-to-centrosome conversion. Nat. Cell Biol. 18:87–99. 10.1038/ncb3274 26595382 PMC4719191

[bib19] Guichard, P., V. Hachet, N. Majubu, A. Neves, D. Demurtas, N. Olieric, I. Fluckiger, A. Yamada, K. Kihara, Y. Nishida, . 2013. Native architecture of the centriole proximal region reveals features underlying its 9-fold radial symmetry. Curr. Biol. 23:1620–1628. 10.1016/j.cub.2013.06.061 23932403

[bib20] Habedanck, R., Y.D. Stierhof, C.J. Wilkinson, and E.A. Nigg. 2005. The Polo kinase Plk4 functions in centriole duplication. Nat. Cell Biol. 7:1140–1146. 10.1038/ncb1320 16244668

[bib21] Hatzopoulos, G.N., M.C. Erat, E. Cutts, K.B. Rogala, L.M. Slater, P.J. Stansfeld, and I. Vakonakis. 2013. Structural analysis of the G-box domain of the microcephaly protein CPAP suggests a role in centriole architecture. Structure. 21:2069–2077. 10.1016/j.str.2013.08.019 24076405 PMC3824074

[bib22] Hsu, W.B., L.Y. Hung, C.J. Tang, C.L. Su, Y. Chang, and T.K. Tang. 2008. Functional characterization of the microtubule-binding and -destabilizing domains of CPAP and d-SAS-4. Exp. Cell Res. 314:2591–2602. 10.1016/j.yexcr.2008.05.012 18586240

[bib23] Izquierdo, D., W.J. Wang, K. Uryu, and M.F. Tsou. 2014. Stabilization of cartwheel-less centrioles for duplication requires CEP295-mediated centriole-to-centrosome conversion. Cell Rep. 8:957–965. 10.1016/j.celrep.2014.07.022 25131205 PMC4152953

[bib24] Kitagawa, D., I. Vakonakis, N. Olieric, M. Hilbert, D. Keller, V. Olieric, M. Bortfeld, M.C. Erat, I. Fluckiger, P. Gonczy, and M.O. Steinmetz. 2011. Structural basis of the 9-fold symmetry of centrioles. Cell. 144:364–375. 10.1016/j.cell.2011.01.008 21277013 PMC3089914

[bib25] Klena, N., M. Le Guennec, A.M. Tassin, H. van den Hoek, P.S. Erdmann, M. Schaffer, S. Geimer, G. Aeschlimann, L. Kovacik, Y. Sadian, . 2020. Architecture of the centriole cartwheel-containing region revealed by cryo-electron tomography. EMBO J. 39:e106246. 10.15252/embj.2020106246 32954513 PMC7667884

[bib26] Kratz, A.S., F. Barenz, K.T. Richter, and I. Hoffmann. 2015. Plk4-dependent phosphorylation of STIL is required for centriole duplication. Biol. Open. 4:370–377. 10.1242/bio.201411023 25701666 PMC4359743

[bib27] Laporte, M.H., D. Gambarotto, E. Bertiaux, L. Bournonville, V. Louvel, J.M. Nunes, S. Borgers, V. Hamel, and P. Guichard. 2024. Time-series reconstruction of the molecular architecture of human centriole assembly. Cell. 187:2158–2174.e19. 10.1016/j.cell.2024.03.025 38604175 PMC11060037

[bib28] Le Guennec, M., N. Klena, D. Gambarotto, M.H. Laporte, A.M. Tassin, H. van den Hoek, P.S. Erdmann, M. Schaffer, L. Kovacik, S. Borgers, . 2020. A helical inner scaffold provides a structural basis for centriole cohesion. Sci. Adv. 6:eaaz4137. 10.1126/sciadv.aaz4137 32110738 PMC7021493

[bib29] Lettman, M.M., Y.L. Wong, V. Viscardi, S. Niessen, S.H. Chen, A.K. Shiau, H. Zhou, A. Desai, and K. Oegema. 2013. Direct binding of SAS-6 to ZYG-1 recruits SAS-6 to the mother centriole for cartwheel assembly. Dev. Cell. 25:284–298. 10.1016/j.devcel.2013.03.011 23673331 PMC3655416

[bib30] Li, S., J.J. Fernandez, W.F. Marshall, and D.A. Agard. 2019. Electron cryo-tomography provides insight into procentriole architecture and assembly mechanism. Elife. 8:e43434. 10.7554/eLife.43434 30741631 PMC6384029

[bib31] Mottier-Pavie, V., and T.L. Megraw. 2009. *Drosophila* Bld10 is a centriolar protein that regulates centriole, basal body, and motile cilium assembly. Mol. Biol. Cell. 20:2605–2614. 10.1091/mbc.e08-11-1115 19321663 PMC2682601

[bib32] Moyer, T.C., K.M. Clutario, B.G. Lambrus, V. Daggubati, and A.J. Holland. 2015. Binding of STIL to Plk4 activates kinase activity to promote centriole assembly. J. Cell Biol. 209:863–878. 10.1083/jcb.201502088 26101219 PMC4477857

[bib33] Nagy, A., L. Kovacs, H. Rangone, J. Fu, M. Ladinsky, and D.M. Glover. 2025. Interactions of N- and C-terminal parts of Ana1 permitting centriole duplication but not elongation. Open Biol. 15:240325. 10.1098/rsob.240325 39904373 PMC11793955

[bib34] Nazarov, S., A. Bezler, G.N. Hatzopoulos, V. Nemcikova Villimova, D. Demurtas, M. Le Guennec, P. Guichard, and P. Gonczy. 2020. Novel features of centriole polarity and cartwheel stacking revealed by cryo-tomography. EMBO J. 39:e106249. 10.15252/embj.2020106249 32954505 PMC7667878

[bib35] Nigg, E.A., and J.W. Raff. 2009. Centrioles, centrosomes, and cilia in health and disease. Cell. 139:663–678. 10.1016/j.cell.2009.10.036 19914163

[bib36] Novak, Z.A., P.T. Conduit, A. Wainman, and J.W. Raff. 2014. Asterless licenses daughter centrioles to duplicate for the first time in Drosophila embryos. Curr. Biol. 24:1276–1282. 10.1016/j.cub.2014.04.023 24835456 PMC4046630

[bib37] Novak, Z.A., A. Wainman, L. Gartenmann, and J.W. Raff. 2016. Cdk1 phosphorylates *Drosophila* Sas-4 to recruit Polo to daughter centrioles and convert them to centrosomes. Dev. Cell. 37:545–557. 10.1016/j.devcel.2016.05.022 27326932 PMC4918730

[bib38] O'Connell, K.F., C. Caron, K.R. Kopish, D.D. Hurd, K.J. Kemphues, Y. Li, and J.G. White. 2001. The *C. elegans* zyg-1 gene encodes a regulator of centrosome duplication with distinct maternal and paternal roles in the embryo. Cell. 105:547–558. 10.1016/s0092-8674(01)00338-5 11371350

[bib39] Ohta, M., T. Ashikawa, Y. Nozaki, H. Kozuka-Hata, H. Goto, M. Inagaki, M. Oyama, and D. Kitagawa. 2014. Direct interaction of Plk4 with STIL ensures formation of a single procentriole per parental centriole. Nat. Commun. 5:5267. 10.1038/ncomms6267 25342035 PMC4220463

[bib40] Panda, P., L. Kovacs, N. Dzhindzhev, A. Fatalska, V. Persico, M. Geymonat, M.G. Riparbelli, G. Callaini, and D.M. Glover. 2020. Tissue specific requirement of *Drosophila* Rcd4 for centriole duplication and ciliogenesis. J. Cell Biol. 219:e201912154. 10.1083/jcb.201912154 32543652 PMC7401805

[bib41] Panda, P., M.S. Ladinsky, and D.M. Glover. 2024. 9-fold symmetry is not essential for centriole elongation and formation of new centriole-like structures. Nat. Commun. 15:4467. 10.1038/s41467-024-48831-y 38796459 PMC11127918

[bib42] Pimenta-Marques, A., T. Perestrelo, P. Reis-Rodrigues, P. Duarte, A. Ferreira-Silva, M. Lince-Faria, and M. Bettencourt-Dias. 2024. Ana1/CEP295 is an essential player in the centrosome maintenance program regulated by Polo kinase and the PCM. EMBO Rep. 25:102–127. 10.1038/s44319-023-00020-6 38200359 PMC10897187

[bib43] Port, F., H.M. Chen, T. Lee, and S.L. Bullock. 2014. Optimized CRISPR/Cas tools for efficient germline and somatic genome engineering in *Drosophila*. Proc. Natl. Acad. Sci. USA. 111:E2967–E2976. 10.1073/pnas.1405500111 25002478 PMC4115528

[bib44] Port, F., N. Muschalik, and S.L. Bullock. 2015. Systematic evaluation of *Drosophila* CRISPR tools reveals safe and robust alternatives to autonomous gene drives in basic research. G3 (Bethesda). 5:1493–1502. 10.1534/g3.115.019083 25999583 PMC4502383

[bib45] Roque, H., A. Wainman, J.H. Richens, K. Kozyrska, A. Franz, and J.W. Raff. 2012. *Drosophila* Cep135/Bld10 maintains proper centriole structure but is dispensable for cartwheel formation. J. Cell Sci. 125:5881–5886. 10.1242/jcs.113506 22976301

[bib46] Ruehle, M.D., S. Li, D.A. Agard, and C.G. Pearson. 2024. Poc1 bridges basal body inner junctions to promote triplet microtubule integrity and connections. J. Cell Biol. 223:e202311104. 10.1083/jcb.202311104 38743010 PMC11094743

[bib47] Saurya, S., H. Roque, Z.A. Novak, A. Wainman, M.G. Aydogan, A. Volanakis, B. Sieber, D.M. Pinto, and J.W. Raff. 2016. *Drosophila* Ana1 is required for centrosome assembly and centriole elongation. J. Cell Sci. 129:2514–2525. 10.1242/jcs.186460 27206860 PMC4958303

[bib48] Sonnen, K.F., L. Schermelleh, H. Leonhardt, and E.A. Nigg. 2012. 3D-structured illumination microscopy provides novel insight into architecture of human centrosomes. Biol. Open. 1:965–976. 10.1242/bio.20122337 23213374 PMC3507176

[bib49] Tang, C.J., R.H. Fu, K.S. Wu, W.B. Hsu, and T.K. Tang. 2009. CPAP is a cell-cycle regulated protein that controls centriole length. Nat. Cell Biol. 11:825–831. 10.1038/ncb1889 19503075

[bib50] Tang, C.J., S.Y. Lin, W.B. Hsu, Y.N. Lin, C.T. Wu, Y.C. Lin, C.W. Chang, K.S. Wu, and T.K. Tang. 2011. The human microcephaly protein STIL interacts with CPAP and is required for procentriole formation. EMBO J. 30:4790–4804. 10.1038/emboj.2011.378 22020124 PMC3243611

[bib51] Tian, Y., C. Wei, J. He, Y. Yan, N. Pang, X. Fang, X. Liang, and J. Fu. 2021. Superresolution characterization of core centriole architecture. J. Cell Biol. 220:e202005103. 10.1083/jcb.202005103 33533934 PMC7863704

[bib52] Tsuchiya, Y., S. Yoshiba, A. Gupta, K. Watanabe, and D. Kitagawa. 2016. Cep295 is a conserved scaffold protein required for generation of a bona fide mother centriole. Nat. Commun. 7:12567. 10.1038/ncomms12567 27562453 PMC5007451

[bib53] van Breugel, M., M. Hirono, A. Andreeva, H.A. Yanagisawa, S. Yamaguchi, Y. Nakazawa, N. Morgner, M. Petrovich, I.O. Ebong, C.V. Robinson, . 2011. Structures of SAS-6 suggest its organization in centrioles. Science. 331:1196–1199. 10.1126/science.1199325 21273447

