# Supplementary figures and images for: Ana1/Cep295 regulates centriole elongation via Cep135 and microtubules
Source: J Cell Biol. 2026 Jul 30;225(10):e202504094. doi: 10.1083/jcb.202504094 (PMC13422256; doi:10.1083/jcb.202504094)

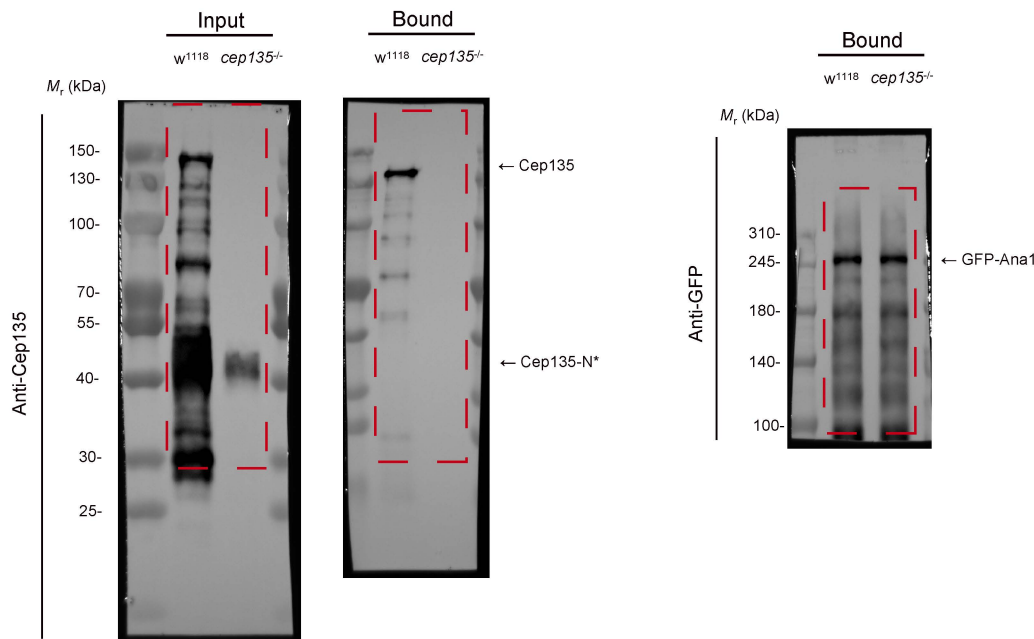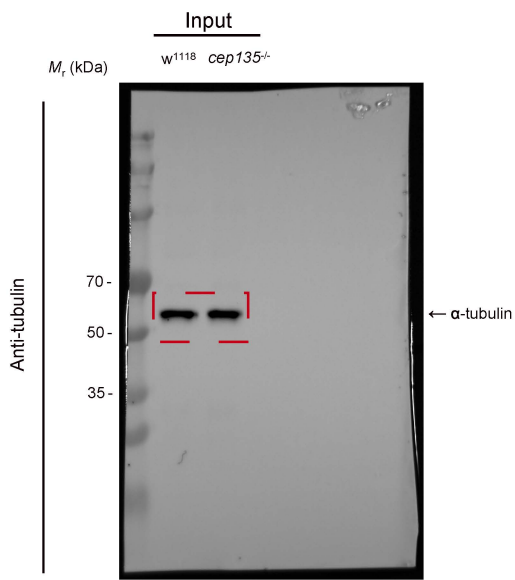

Supplement: SourceData F1 — is the source file for Fig. 1. [file jcb_202504094_sourcedataf1.pdf]

Figure 2

A

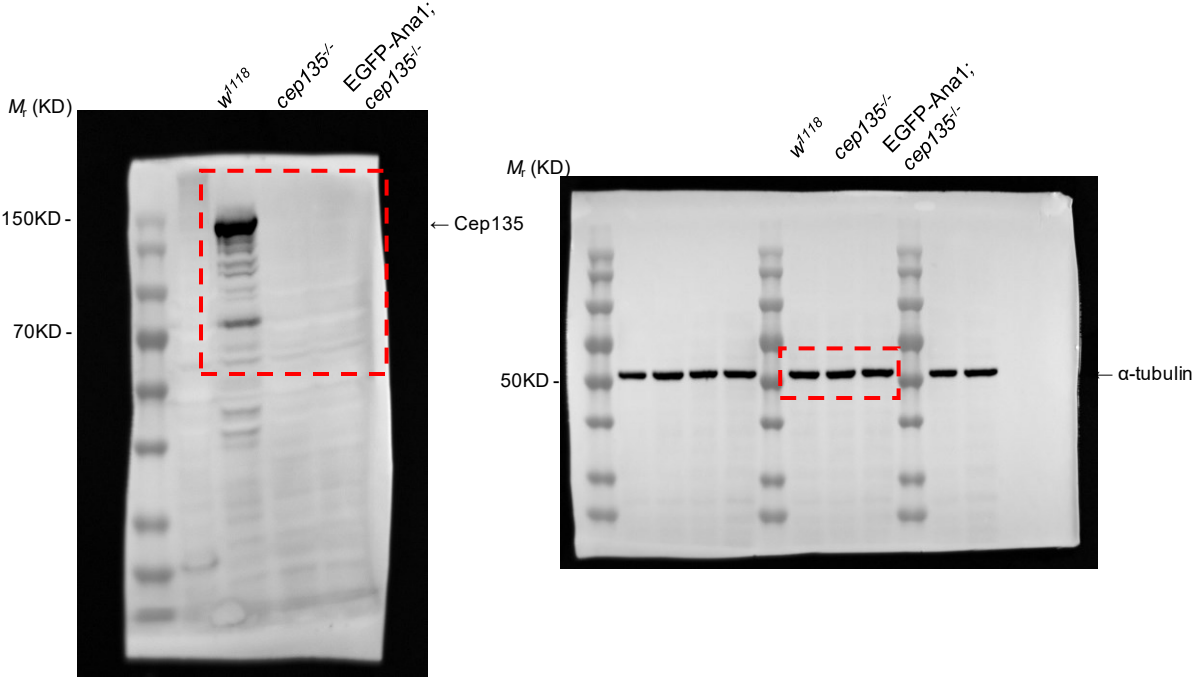

Supplement: SourceData F2 — is the source file for Fig. 2. [file jcb_202504094_sourcedataf2.pdf]

B

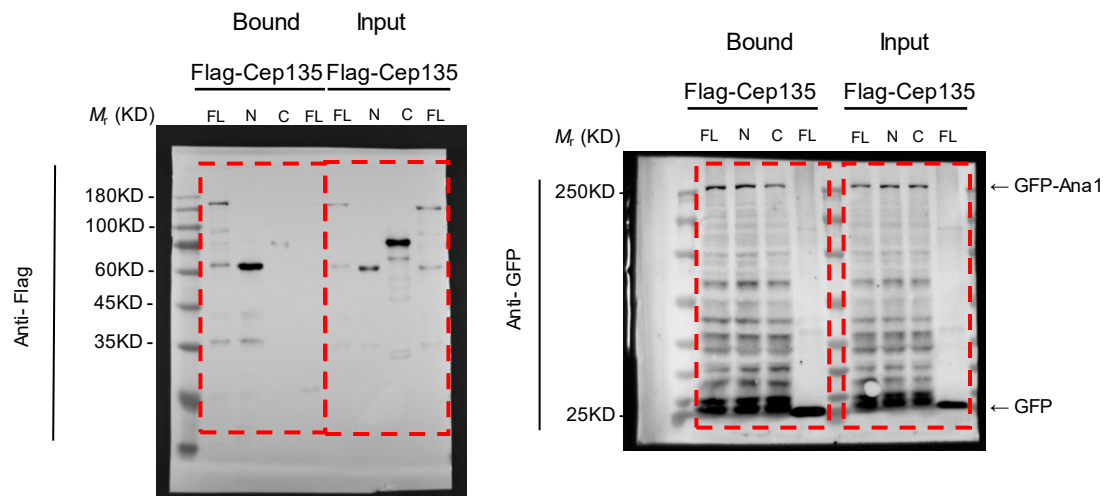

C

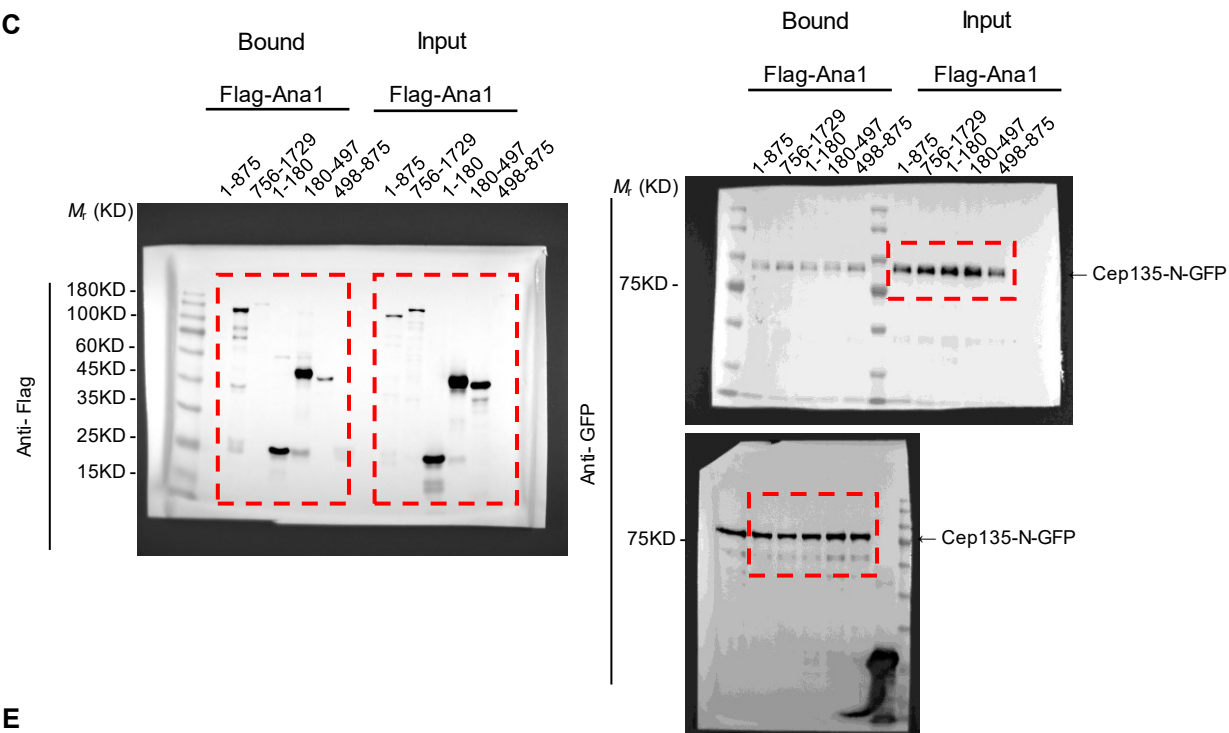

E

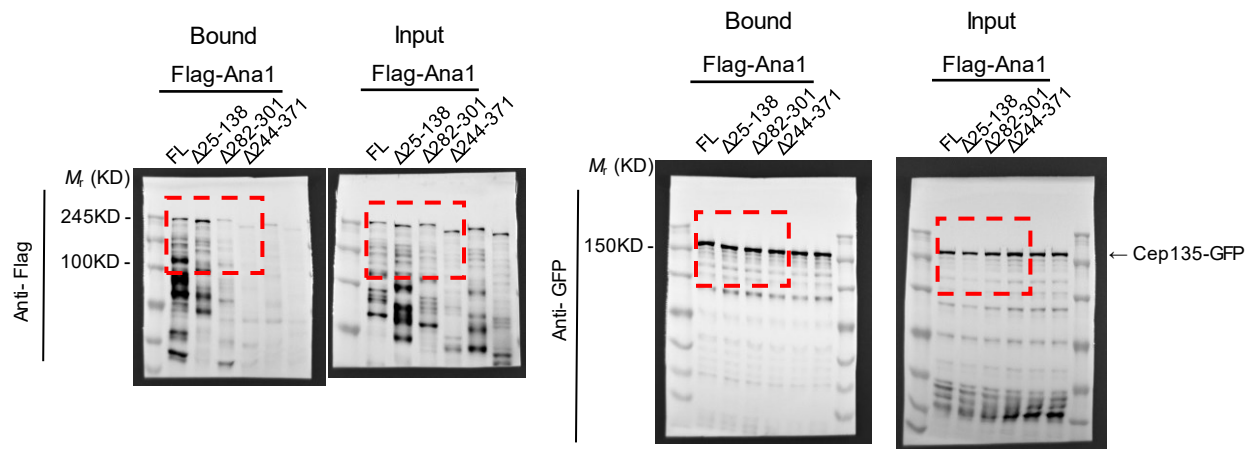

Supplement: SourceData F3 — is the source file for Fig. 3. [file jcb_202504094_sourcedataf3.pdf]

**A**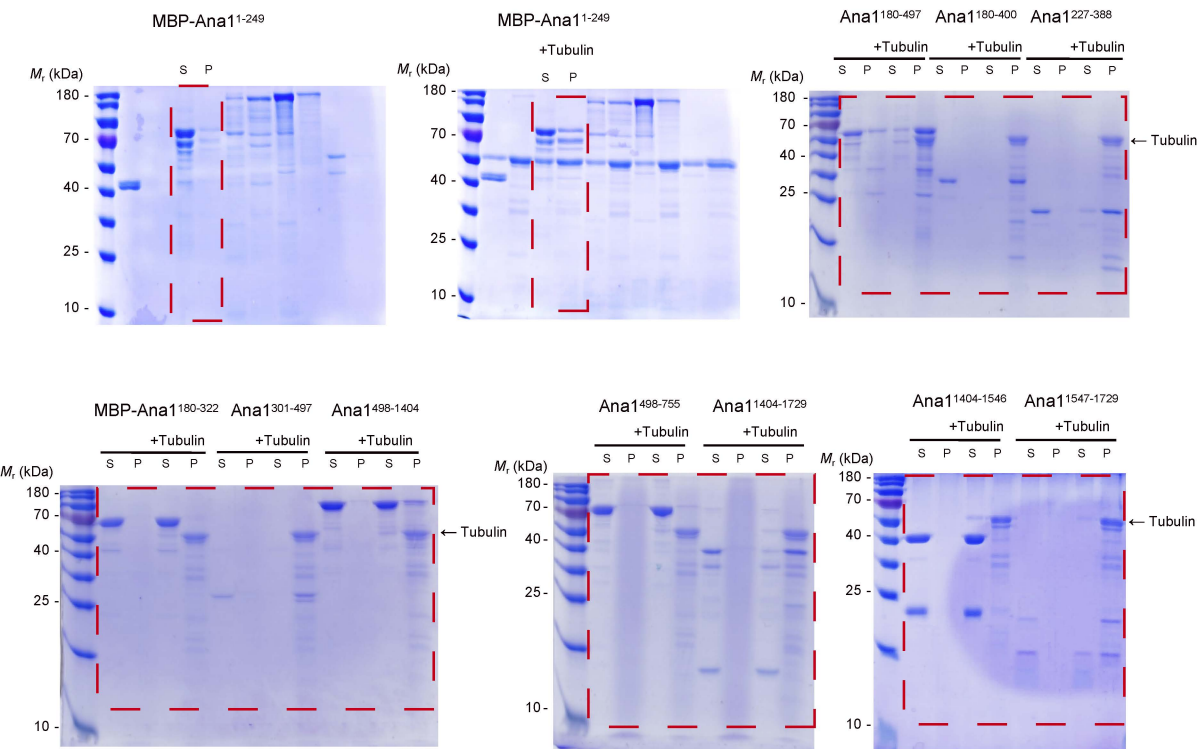**D**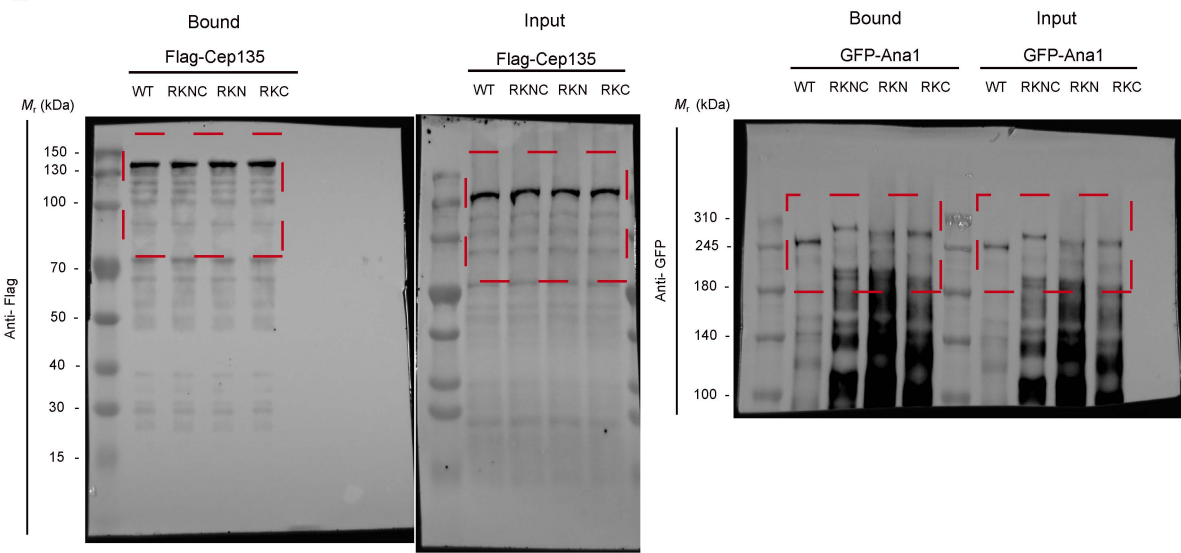

Supplement: SourceData F4 — is the source file for Fig. 4. [file jcb_202504094_sourcedataf4.pdf]

Figure 5

A

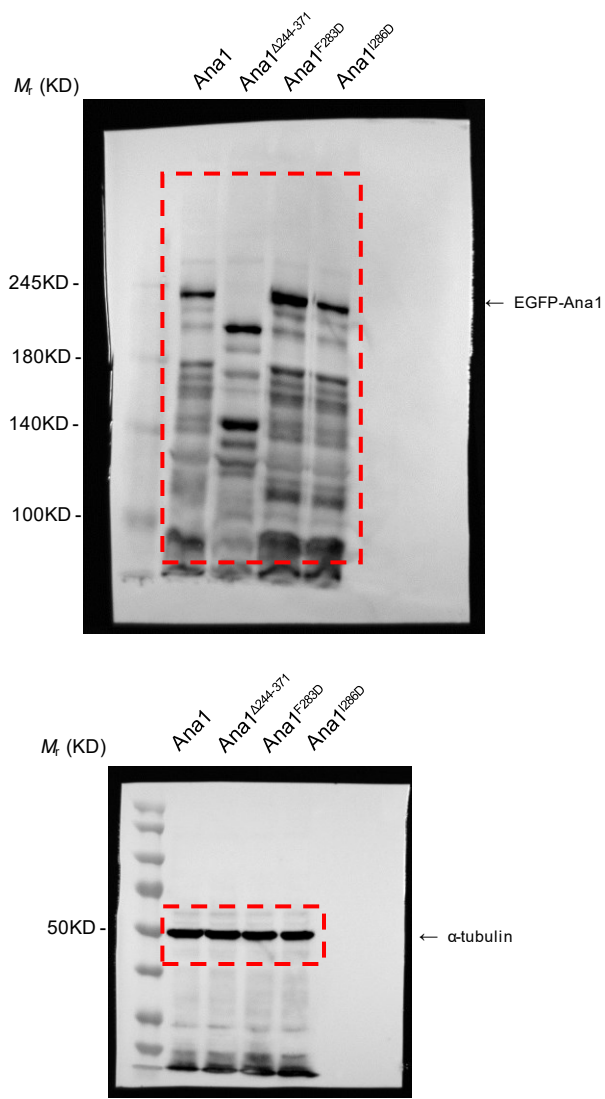

Supplement: SourceData F5 — is the source file for Fig. 5. [file jcb_202504094_sourcedataf5.pdf]

Figure 7

A

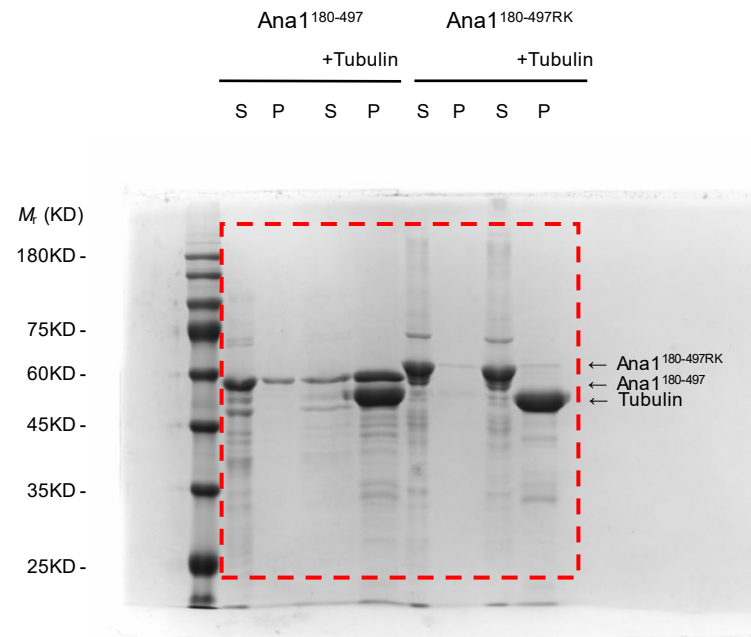

B

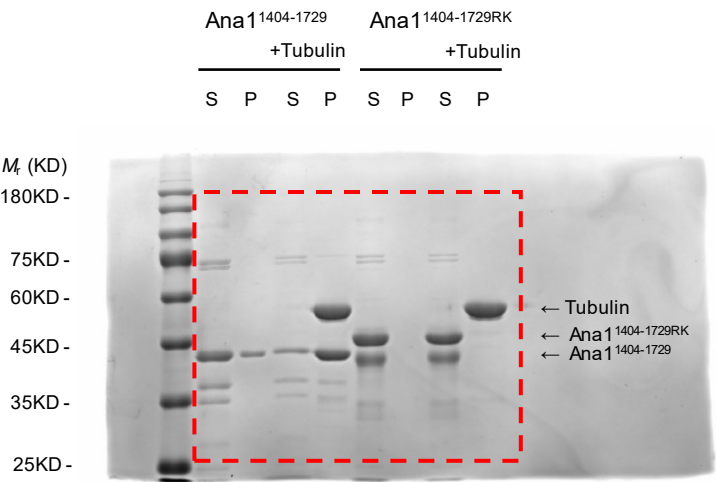

Supplement: SourceData F7 — is the source file for Fig. 7. [file jcb_202504094_sourcedataf7.pdf]

# Figure 8

A

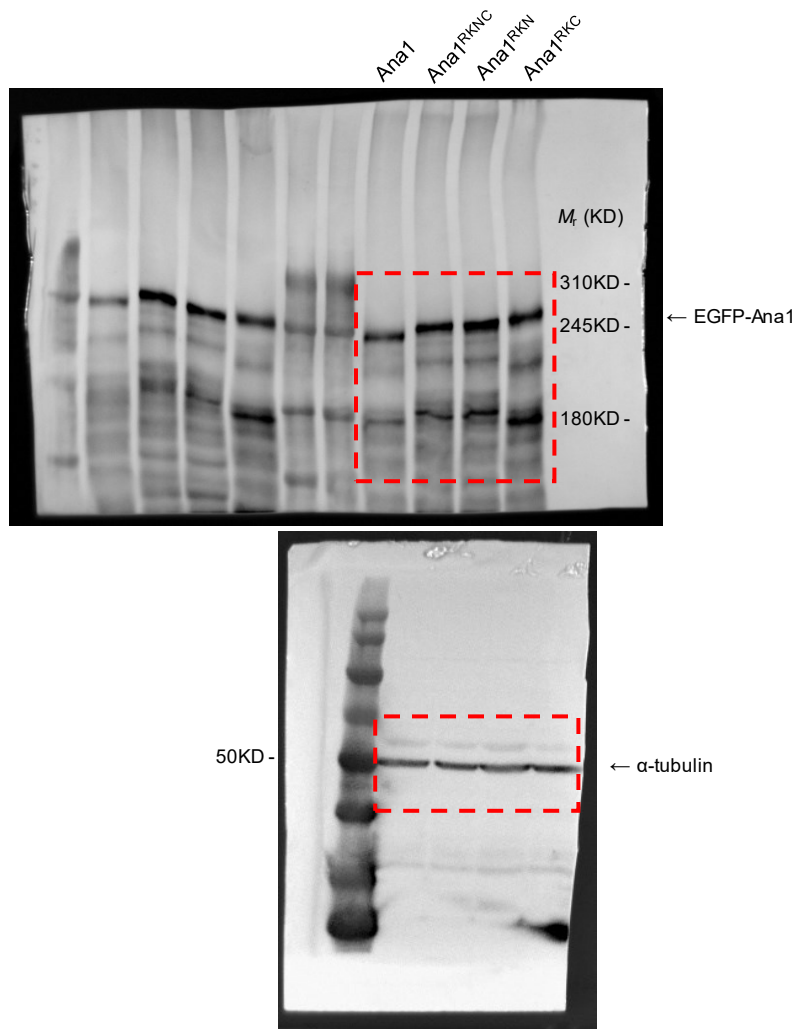

Supplement: SourceData F8 — is the source file for Fig. 8. [file jcb_202504094_sourcedataf8.pdf]

Figure S1

C

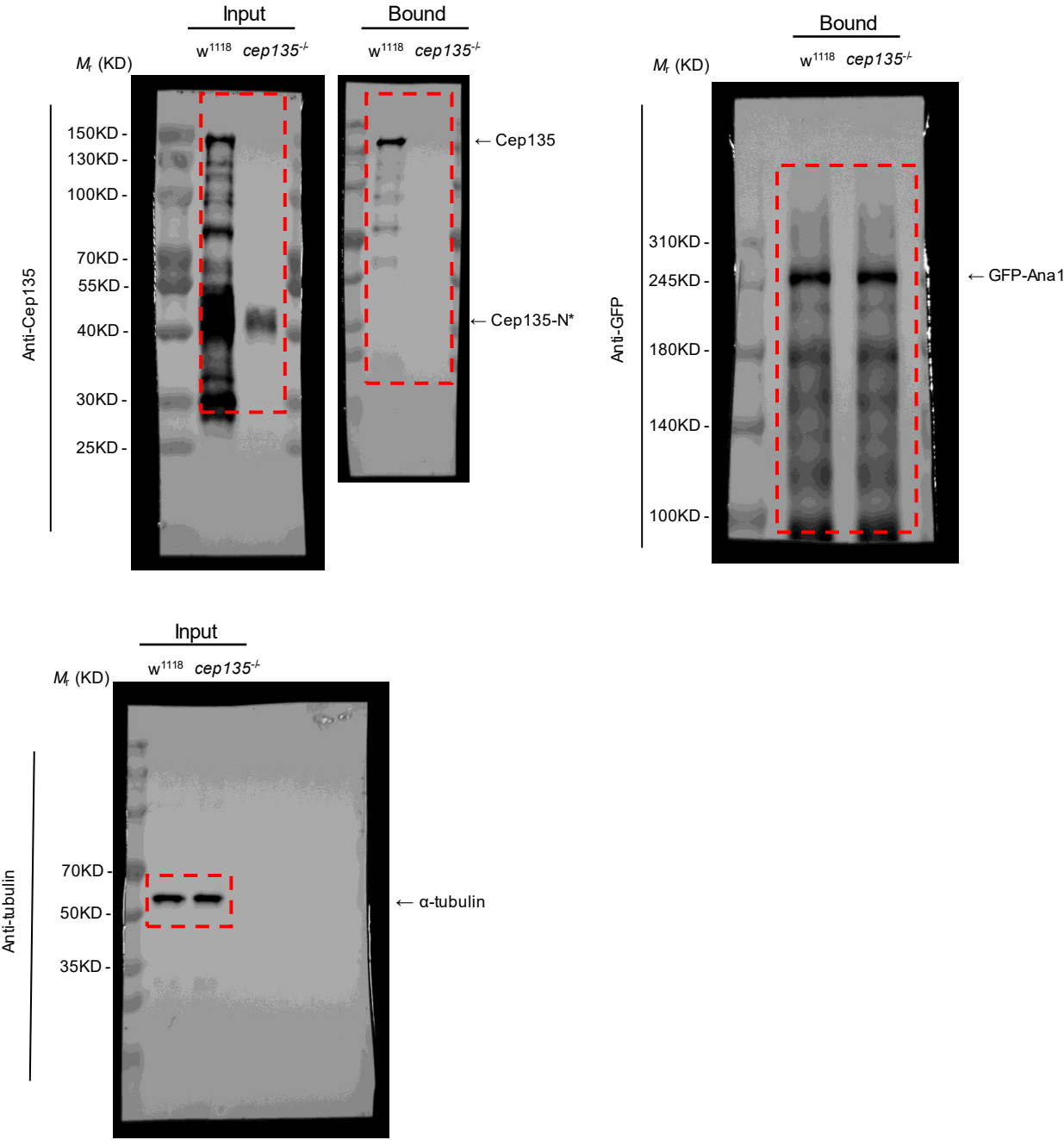

Supplement: SourceData FS1 — is the source file for Fig. S1. [file jcb_202504094_sourcedatafs1.pdf]

# Figure S3

**A**

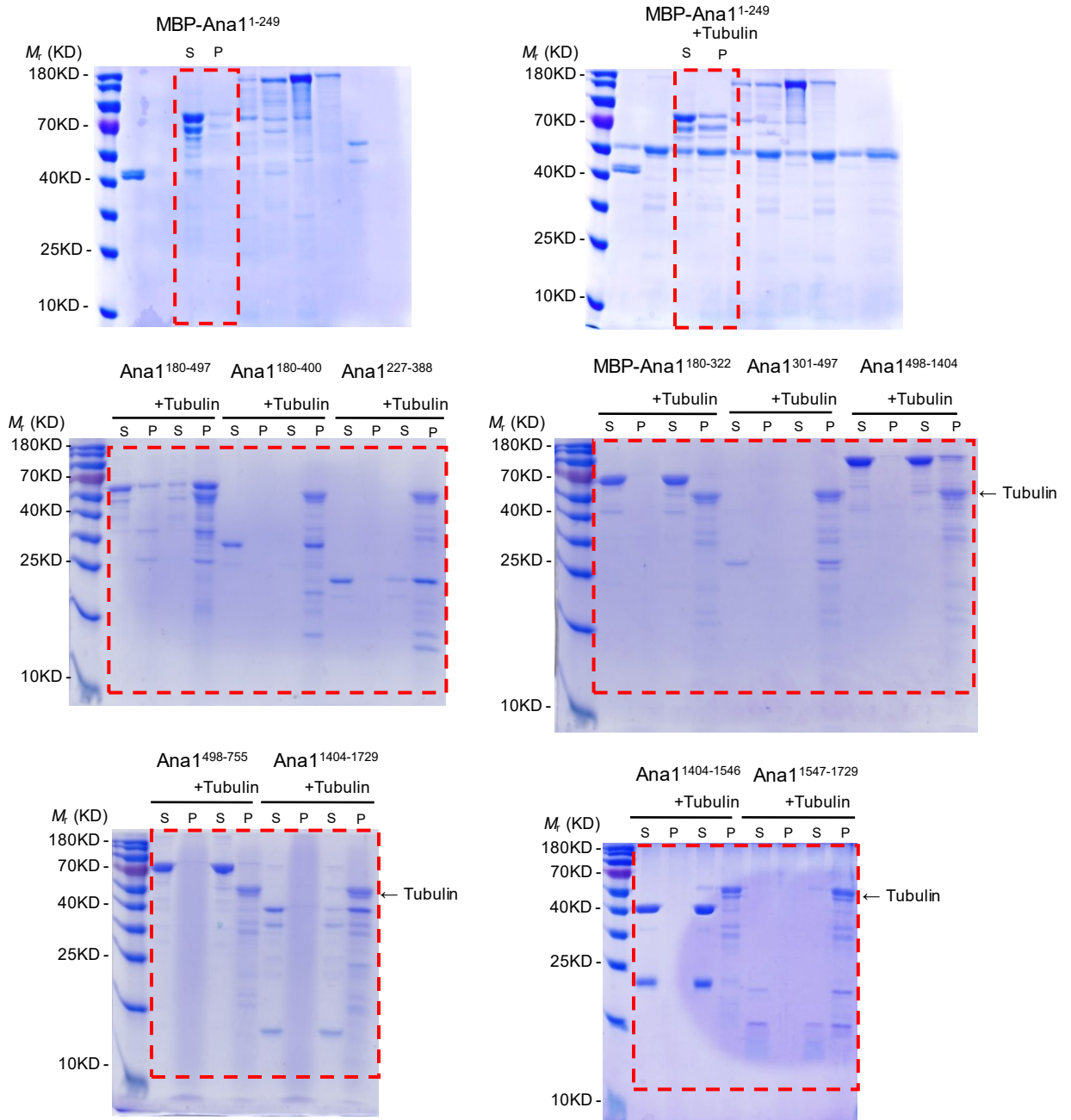

**D**

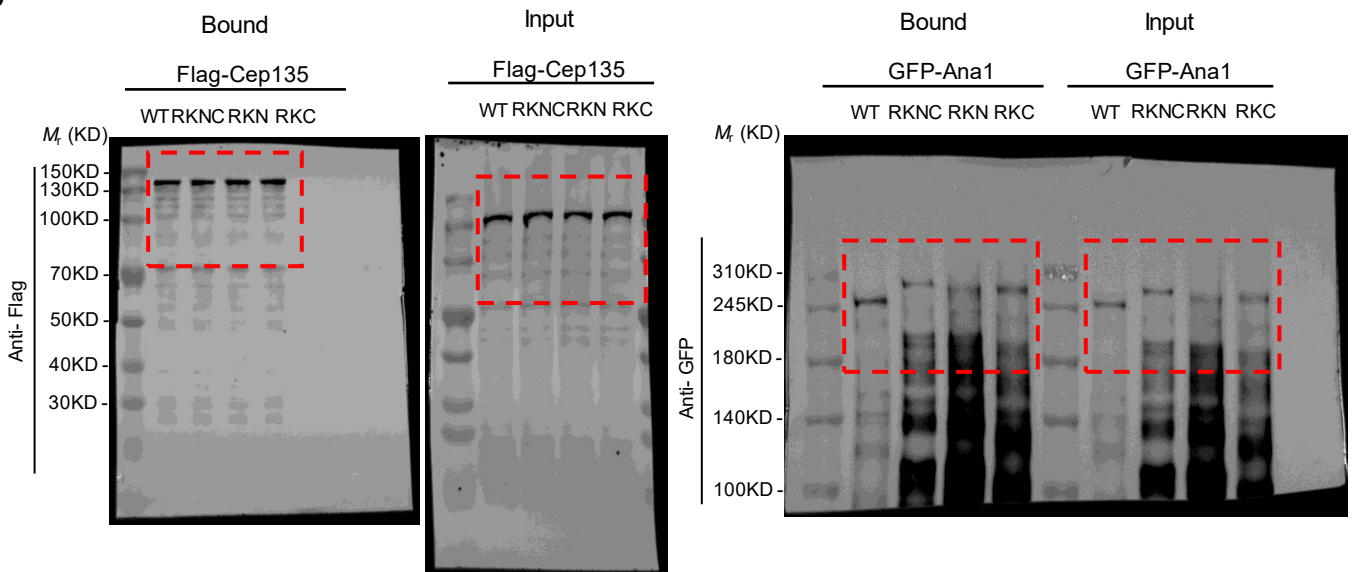

Supplement: SourceData FS4 — is the source file for Fig. S4. [file jcb_202504094_sourcedatafs4.pdf]

# Figure S4

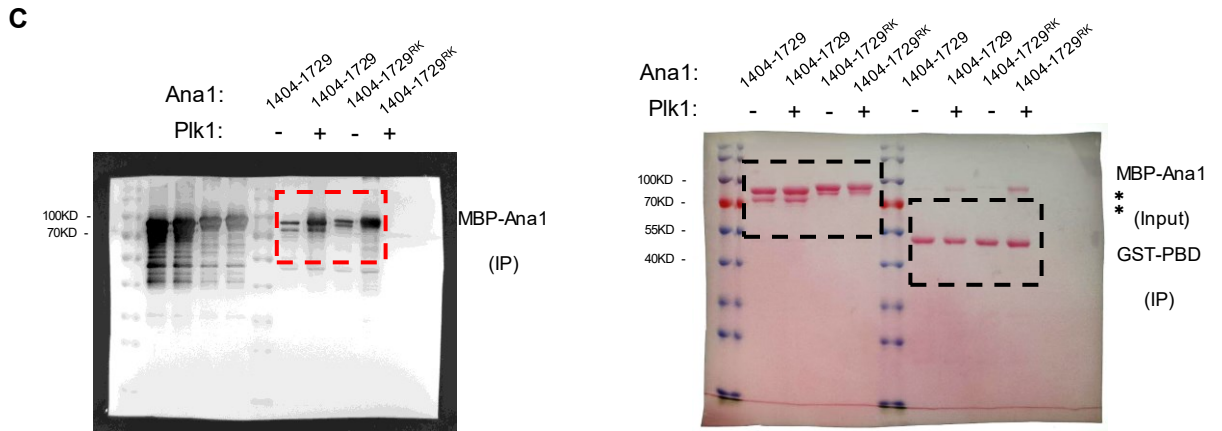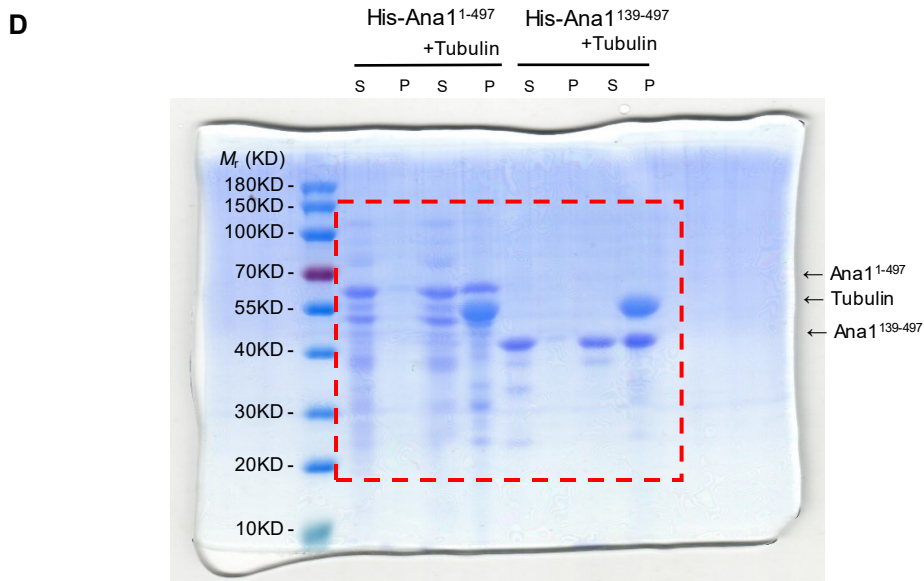

Supplement: SourceData FS5 — is the source file for Fig. S5. [file jcb_202504094_sourcedatafs5.pdf]
